# ATP1A1 enhances porcine reproductive and respiratory syndrome virus type 2 attachment and internalization

**DOI:** 10.1128/mbio.03896-25

**Published:** 2026-03-03

**Authors:** Haotian Yang, Bicheng Li, Xudong Yin, Aldryan Cristianto Pratama, Fang He

**Affiliations:** 1 MOA Key Laboratory of Animal Virology, Zhejiang University Center for Veterinary Sciences, Hangzhou, China; 2 Institute of Preventive Veterinary Medicine, College of Animal Sciences, Zhejiang University 12377, Hangzhou, China; 3 ZJU-Xinchang Joint Innovation Centre (TianMu Laboratory), Xinchang, Zhejiang, China; 4 Zhejiang Provincial Engineering Research Center of Animal Biological Products, Zhejiang University Center for Veterinary Sciences, Hangzhou, China; Washington University in St. Louis School of Medicine, St. Louis, Missouri, USA

**Keywords:** ATP1A1, PRRSV, virus entry, endocytotic pathway, virus blocking strategy

## Abstract

Na^+^-K^+^-ATPase alpha subunit 1 (ATP1A1) is the main functional part of the sodium pump. In addition to the well-known role in ion transport, it also acts as a signal transducer. Porcine reproductive and respiratory syndrome virus type 2 (PRRSV-2) utilizes multiple entry factors and pathways to initiate infection, posing a significant challenge to the global swine industry. However, the molecules conferring PRRSV-2 infection have not been fully characterized. Here, ATP1A1 is identified as a novel factor in PRRSV-2 attachment and internalization. ATP1A1 formed clusters in the plasma membrane very early following PRRSV-2 infection and co-internalizes with virions. Knockdown of ATP1A1 significantly suppressed PRRSV-2 infection by reducing viral attachment, and the specific chemical ligands, ouabain and PST2238, effectively reduced viral internalization without affecting viral attachment, leading to decreased viral infection. Mechanically, ATP1A1-Src signaling-dependent activation of EGFR and caveolin-1 was required for efficient PRRSV-2 uptake through macropinocytosis and caveolae/raft-mediated endocytosis. Furthermore, internalized virions were subsequently trafficked to ATP1A1/CD163-positive early endosomes, where uncoating occurs. In detail, PRRSV glycoprotein 4 (GP4), a major determinant for viral cellular tropism, was found to interact with the fourth extracellular region (ER4) of ATP1A1, dependent on its C-terminus. A synthetic ATP1A1-ER4 peptide inhibited PRRSV-2 replication by competitively reducing viral attachment and internalization in a dose-dependent manner. Most importantly, a specific nanobody targeting ATP1A1-ER4 provided broad inhibition against various PRRSV-2 lineages both in PAMs and Marc-145 cells. Collectively, these results elucidate that ATP1A1 is important for PRRSV-2 attachment and internalization, offering a potential target for the development of antiviral treatments.

**IMPORTANCE:**

PRRSV continues to cause severe financial losses to the global swine industry. It is feasible to develop safe and effective antiviral strategies based on the initial step of viral infection, that is, the recognition of the virus by the cellular entry factors. However, the interactions between PRRSV and host factors initiating viral attachment and internalization are not fully understood yet. In this study, ATP1A1 was identified to promote both PRRSV-2 attachment and internalization through macropinocytosis and caveolae/raft-dependent endocytosis. These findings reveal an unrecognized entry mechanism of PRRSV-2 and provide novel insights for the development of antiviral drugs and vaccines against the virus.

## INTRODUCTION

Porcine reproductive and respiratory syndrome virus (PRRSV) is one of the major threats that may cause severe respiratory disease in infected piglets and reproductive failure in pregnant sows, causing huge economic losses to global pig industry (1). PRRSV belongs to the genus Betaarterivirus in the family Arteriviridae and order Nidovirales (2). PRRSV contains a positive-strand RNA genome with a length of 15 kb, encoding eight structural proteins, including glycoproteins (GPs) GP2a, GP3, GP4, GP5, nucleocapsid (N) protein, envelope (E) protein, matrix (M) protein, ORF5a protein, and at least 16 nonstructural proteins (3). PRRSV is typically categorized into two species: PRRSV-1 (European-like isolate) and PRRSV-2 (North America-like isolate). Since 2006, PRRSV-2 has rapidly spread to become the primary epidemic strain due to its higher pathogenicity and more widespread emergence. It is worth noting that during recent years, PRRSV-2 outbreaks exhibited new epidemiological characteristics, including increased diversity of viral lineages, more frequent recombination events, and recovered virulence, impeding the process of PRRS prevention and control (4).

The first step of viral infection is binding to attachment factors, including proteins, carbohydrates, or lipids on the cell surface, followed by interacting with entry factors to mediate efficient invasion. Conceptually, entry factors can be broadly divided into internalization factors and fusogenic factors, based on their primary roles during entry. Internalization factors can also mediate further viral attachment and uptake by triggering endocytic pathways such as clathrin-mediated endocytosis (CME), macropinocytosis, and caveolae/lipid raft-dependent endocytosis and accompanying virions into host cells (5). In contrast, fusogenic factors act at a later stage to promote membrane fusion, viral uncoating, and genome release, frequently within endosomal compartments (6). Productive infection by many enveloped viruses requires the coordinated action of both factor types rather than a single entry molecule.

Na^+^/K^+^-ATPase is a P-type ATPase located in the plasma membrane and transports sodium and potassium ions to maintain intracellular electrolyte and fluid balance. Besides the high physiological significance of Na^+^/K^+^-ATPase as an ion pump, research in recent years elucidated the vital role of Na^+^/K^+^-ATPase as a signal transducer, which mainly functions via Na^+^/K^+^-ATPase alpha subunit 1 (ATP1A1) (7). ATP1A1 is the major subunit of Na^+^/K^+^-ATPase, widely expressed in eukaryotic cells with 10 transmembrane helices. It is well-known that Src kinase is a key mediator of the ATP1A1-related signaling pathway. Although ATP1A1 itself lacks a recognized cytoplasmic signaling domain, it physically interacts with Src kinase through its cytoplasmic tail. This binding induces a conformational change in Src kinase, exposing its kinase domain and leading to its activation via autophosphorylation (8). Once activated, Src kinase can initiate several downstream signaling cascades, ultimately culminating in the induction of endocytosis through various mechanisms, including CME (9), caveolae/raft-mediated endocytosis (10), or macropinocytosis (11). A series of studies have reported that ATP1A1 plays a vital role during viral invasion. For instance, ATP1A1 facilitates cellular entry of not only various coronaviruses including Middle East respiratory syndrome (MERS)-CoV, porcine epidemic diarrhea virus (PEDV), feline infectious peritonitis virus (FIPV), and murine hepatitis coronavirus (MHV) (12, 13) but also other viruses such as vesicular stomatitis virus (VSV) (12), respiratory syncytial virus (RSV) (14), and white spot syndrome virus (WSSV) (15).

Nowadays, although PRRSV exhibits high genetic diversity, it shows quite restricted cellular tropism, including porcine alveolar macrophages (PAMs) *in vivo* (16), and African green monkey kidney epithelial cell line MA-104 and its derivative, Marc-145, are susceptible to PRRSV infection *in vitro* (17). Despite its narrow host range, the process of PRRSV entry into the target cells is a complex event mediated by specific host factors. To date, at least 12 molecules have been demonstrated as PRRSV invasion factors, including HS, Sn, DC-SIGN, vimentin, CD151, MYH9, HSPA8, TIM 1/4, Siglec-10, Syndecan-4, FcRn, and CD163 (18–22). In the classical invasion model of PRRSV, HS is viewed as a critical factor for mediating the initial attachment, subsequently facilitating Sn-mediated internalization for successful infection. Simultaneously, other factors, such as MYH9, HSPA8 (a molecular chaperone), and others, cooperatively facilitate this process. Subsequently, FcRn facilitated virion uncoating and genome release. During the whole process, CD163 has been confirmed as an indispensable receptor for PRRSV infection by gene knockout experiments *in vivo* (22) and proposed to participate in viral internalization and uncoating steps (23), although the detailed mechanism of CD163-related uncoating still needs to be further determined. However, PRRSV can also bind to and even further internalize into non-permissive cells (24, 25). Moreover, HS is not absolutely required for PRRSV invasion (26). The function of Sn in PRRSV invasion is also controversial. Sn is a macrophage-specific factor mediating viral internalization, which is not naturally expressed in Marc-145 cells (18). Moreover, even one previous study reported that an intact Sn is not required for PRRSV infection *in vivo* (27). These findings strongly suggest the involvement of another unidentified molecule(s) in PRRSV initial entry process. Recently, one study has implicated the critical role of Src kinase in PRRSV early entry process (28). However, the vital upstream initiating molecule remains to be identified. Given the activating effect of ATP1A1 on Src-induced signaling cascades, ATP1A1 may also participate in PRRSV entry.

In this study, the role of ATP1A1 in PRRSV-2 infection was first explored, and further analysis demonstrated that it is involved in PRRSV-2 attachment and internalization via macropinocytosis and caveolae/raft-mediated endocytosis pathways. An interaction identified between the fourth extracellular region of ATP1A1 and GP4-C terminus initiates this entry process. Most importantly, a specific nanobody blocking ATP1A1 provides broad inhibition against various PRRSV-2 lineages in PAMs and Marc-145 cells. These findings revealed a novel mechanism by which PRRSV-2 invades host cells and paved the way for the development of novel strategies for PRRSV-2 prevention and control.

## RESULTS

### ATP1A1 is important for PRRSV-2 attachment, leading to a co-entry process with virions

**Fig 1:**
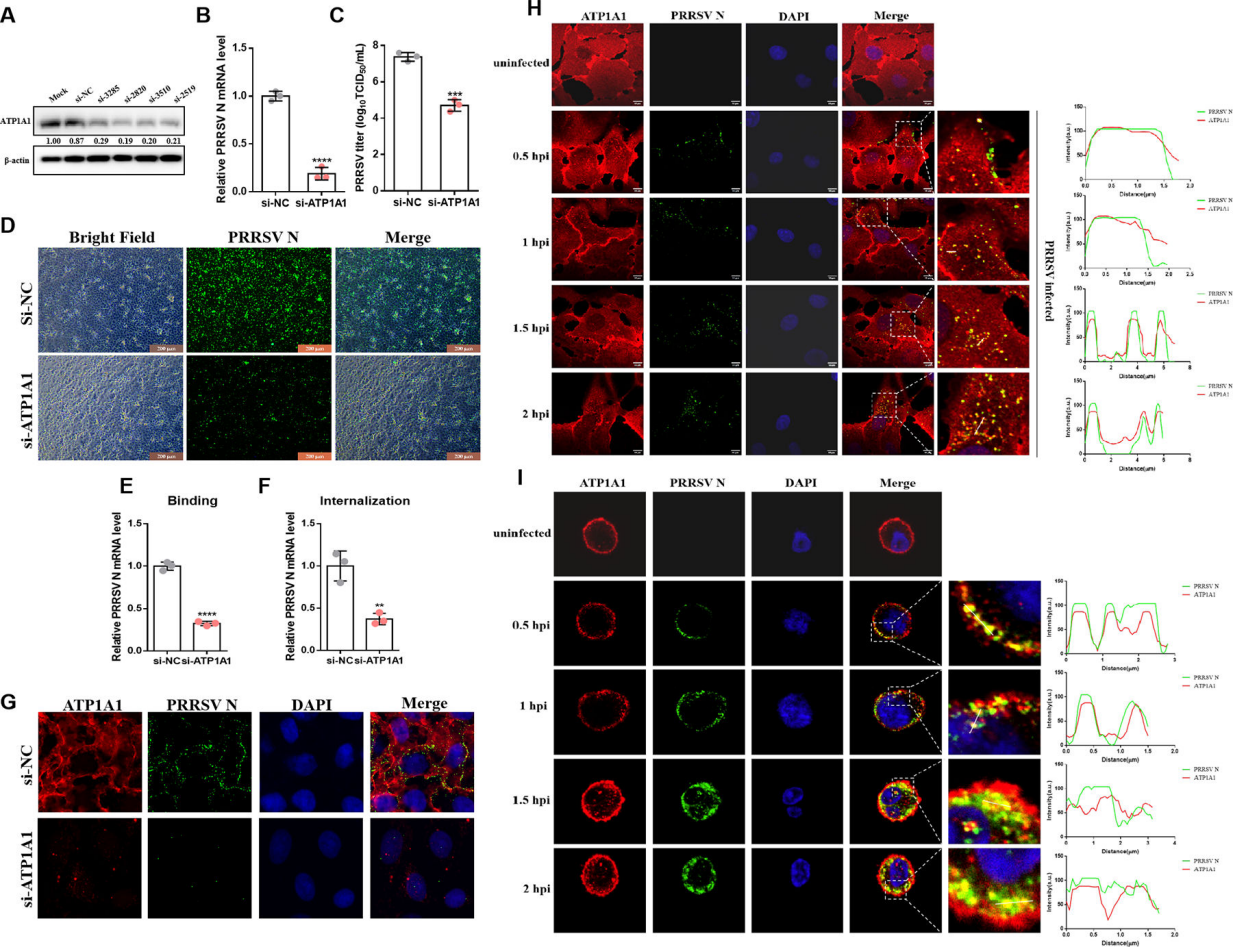
ATP1A1 is important for PRRSV-2 attachment, leading to a co-entry process with virions. (**A**) The knockdown efficiency of siRNAs targeting ATP1A1. Four siRNAs that knock down Chlorobacterium sabaeus ATP1A1 were constructed and transfected into Marc-145 cells, with si-NC used as a negative control. At 48 h post-transfection, the cell lysates were collected to detect the knockdown efficiency via western blotting analysis. (**B–D**) PRRSV-2 replication level in ATP1A1-knockdown Marc-145 cells. Cells were transfected with si-ATP1A1 or si-NC for 48 h and then infected with the PRRSV HuN4-F112 strain (MOI = 1) infection. (**B**) qRT-PCR analysis of the mRNA level of PRRSV N at 24 hpi. (**C**) Cell culture supernatants were collected to determine the viral titers at 48 hpi. (**D**) PRRSV N protein (green) was assessed via IFA at 48 hpi. (**E and F**) ATP1A1 knockdown significantly reduced PRRSV-2 attachment and internalization. Marc-145 cells were transfected with si-ATP1A1 or si-NC for 48 h. For the viral attachment assay, cells were infected with the PRRSV HuN4-F112 strain (MOI = 50) and incubated at 4°C for 2 h. Then, the cells were washed with cold PBS for six times, and the cell lysates were collected to detect viral RNA abundance via qRT-PCR (**E**). For the viral internalization assay, cells were infected with the PRRSV HuN4-F112 strain (MOI = 50) and incubated at 4°C for 2 h. The cells were washed with cold PBS six times and then incubated at 37°C for 2 h to complete virus internalization. Then, the cells were washed again for 6 times with acidic PBS (pH = 2.0) to remove the uninternalized virus particles. After that, cell lysates were collected to detect viral RNA abundance via qRT-PCR (**F**). (**G**) Confocal images of PRRSV-2 binding in ATP1A1-knockdown cells. Cells were transfected with siRNA targeting ATP1A1 or si-NC for 48 h and then infected with the PRRSV HuN4-F112 strain (MOI = 50) at 4°C for 2 h. The cells were then fixed and stained with rabbit anti-ATP1A1 pAb (red) and mouse anti-PRRSV N mAb (green). Nuclei were labeled with DAPI (blue). (**H and I**) Colocalization of ATP1A1 and PRRSV-2 particles. Marc-145 cells with the PRRSV HuN4-F112 strain (MOI = 50) or PAMs with PRRSV HNXX16 strain (MOI = 10) infection were collected at the indicated time points. Then, the cells were fixed and stained with rabbit anti-ATP1A1 pAb (red) and mouse anti-PRRSV N mAb (green). Nuclei were labeled with DAPI (blue). Statistical analysis was performed via t tests, and data were presented as the means ± SDs. Significant differences were indicated as follows: * (*P* < 0.05), ** (*P* < 0.01), *** (*P* < 0.001), and **** (*P* < 0.0001).

ATP1A1 has been demonstrated to participate in the entry stage of various viruses (12–15). To investigate whether ATP1A1 is involved in PRRSV-2 infection, four specific small-interfering RNAs (siRNAs) targeting monkey ATP1A1 were synthesized. As shown in Fig. 1A, compared to the control group (siRNA-NC), siRNA-2820 showed the strongest efficiency in decreasing ATP1A1 protein expression. Hence, the siRNA-2820 was selected for further experiments. Subsequently, the effect of ATP1A1 knockdown on PRRSV-2 replication was detected in Marc-145 cells. At 48 h post-siRNA-2820 transfection, followed by PRRSV HuN4-F112 infection, significant reductions in PRRSV N mRNA level (80%–90%) and viral titers (about 100-fold) were detected (Fig. 1B and C). By immunofluorescence assay (IFA), ATP1A1 knockdown significantly reduced the fluorescent signal of PRRSV N protein compared to the control group (Fig. 1D). These results suggest that ATP1A1 acts as a positive host factor benefiting PRRSV-2 infection.

Furthermore, to investigate which stage(s) of PRRSV-2 replication are affected by ATP1A1, PRRSV-2 attachment and internalization were first assayed in ATP1A1 knockdown cells and quantified by RT-qPCR. Surprisingly, knockdown of ATP1A1 significantly decreased the amount of PRRSV-2 attachment and internalization into Marc-145 cells (Fig. 1E and F). The laser confocal images revealed that the number of virions retained on the cell membrane after incubation at 4°C for 2 h was significantly reduced following ATP1A1 knockdown, indicating decreased viral attachment (Fig. 1G). Furthermore, laser confocal microscopy was carried out with Marc-145 cells and primary porcine alveolar macrophages (PAMs, the primary targets of PRRSV-2 i*n vivo*) to visualize the subcellular localization of ATP1A1 and PRRSV-2 virions. Interestingly, following infection with PRRSV-2, some clusters of ATP1A1 were observed as early as 0.5 h post-infection (hpi) and co-localized with virions, whereas these clusters were not evident in uninfected cells. With the virions gradually internalized into cells, the co-localization of ATP1A1 clusters and virions continued to be more prominent and numerous, as shown in the period from 1 to 2 hpi (Fig. 1H and I). Taken together, these results indicated that ATP1A1 participates in PRRSV-2 attachment to susceptible cells, including Marc-145 cells and PAMs, leading to a co-entry process with virions.

### ATP1A1 participates in PRRSV-2 internalization

**Fig 2:**
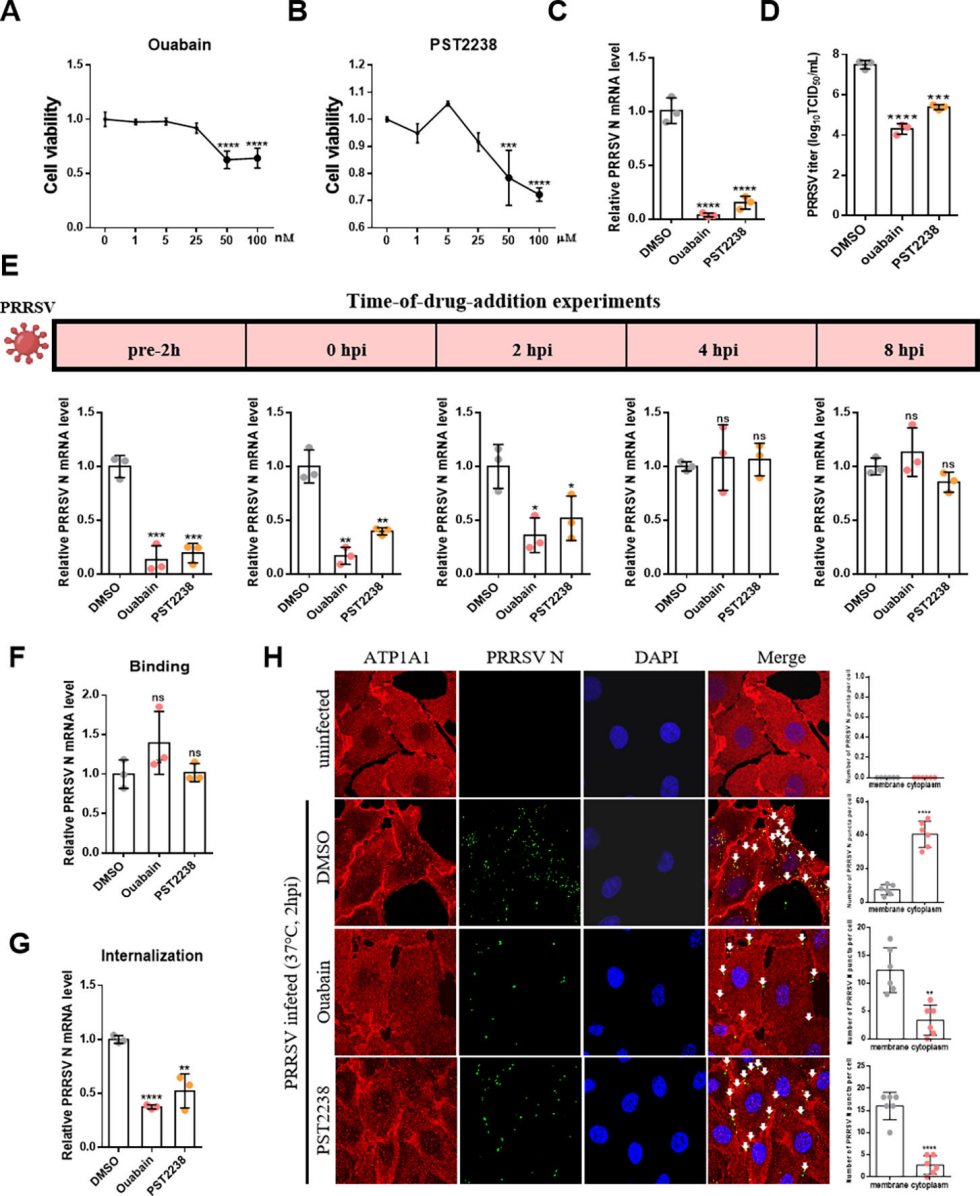
ATP1A1 participates in PRRSV-2 internalization. (**A and B**) The cytotoxicity of ouabain and PST2238. Marc-145 cells were treated with 1, 5, 25, 50, and 100 nM ouabain and 1, 5, 25, 50, and 100 µM PST2238 for 48 h. The treated cells were then analyzed using the CCK-8 kit. (**C and D**) The effect of ouabain (25 nM) and PST2238 (25 µM) on PRRSV-2 infection. Marc-145 cells were pre-treated with two inhibitors for 2 h, followed by PRRSV HuN4-F112 strain (MOI = 1) infection (with compounds present throughout). At 24 hpi, the cell lysates were collected to detect viral RNA abundance via qRT-PCR (**C**), and the supernatants were collected to detect viral titers at 48 hpi (**D**). (**E**) Time-of-drug-addition experiment. Marc-145 cells were treated with ouabain (25 nM) and PST2238 (25 µM) at the indicated times pre- or post-infection. The cell lysates were collected at 10 hpi to detect viral RNA abundance via qRT-PCR. (**F and G**) The effect of ouabain (25 nM) and PST2238 (25 µM) on PRRSV-2 attachment and internalization. Marc-145 cells were pre-treated with two inhibitors for 2 h, then the viral attachment (**F**) and internalization (**G**) assays were conducted in Marc-145 cells. (**H**) IFA analysis of PRRSV-2 internalization in Marc-145 cells pre-treated with ouabain (25 nM) and PST2238 (25 µM). Marc-145 cells were pre-treated with two inhibitors for 2 h, followed by the PRRSV HuN4-F112 strain (MOI = 50) at 37°C for 2 h. After that, the cells were fixed and stained with rabbit anti-ATP1A1 pAb (red) and mouse anti-PRRSV N mAb (green). Nuclei were labeled with DAPI (blue). The subcellular localization of viral particles was detected by confocal assay (left), and the number of PRRSV N puncta (located in the membrane and cytoplasm) per cell was counted (in total, six individual cells) (right). Statistical analysis was performed via t tests, and data were presented as the means ± SDs. Significant differences were indicated as follows: ns (*P* > 0.05), * (*P* < 0.05), ** (*P* < 0.01), *** (*P* < 0.001), and **** (*P* < 0.0001).

Due to the impaired PRRSV attachment on cells after ATP1A1 knockdown, the level of viral internalization was also influenced. Additional experiments are performed to confirm the role of ATP1A1 in PRRSV-2 internalization. Here, two well-known chemical ligands for ATP1A1 signaling, ouabain and PST2238, were selected to test the effects on PRRSV-2 infection. The cytotoxicity of serial dilutions of ouabain and PST2238 on Marc-145 cells was evaluated using the CCK-8 kit (Fig. 2A and B). Based on these results, concentrations of 25 nM ouabain and 25 μM PST2238 were chosen for having a minimal impact on cell viability (less than 10% reduction). As shown in Fig. 2C and D, both ouabain and PST2238 effectively reduced viral RNA copy number and the yield of progeny PRRSV-2. Ouabain exhibited the most powerful antiviral effect, decreasing the viral RNA copy number by about 95% and diminishing the virus yield by almost 1,000-fold relative to the DMSO control. While also effective, PST2238 showed corresponding reductions of 85% (viral RNA) and approximately 100-fold (virus yield).

To investigate the stage of PRRSV-2 infection inhibited by these two compounds, a “time of addition” experiment was subsequently performed. According to a previous study, the first replication cycle of PRRSV-2 occurs within 8 h (29). Hence, we conducted drug treatment at different time points within 8 h after infection and collected samples at 10 hpi to detect the viral RNA copy number by RT-qPCR. As shown in Fig. 2E, the strongest suppression of viral replication occurred when the inhibitors were pre-treated for 2 h before infection. As the timing of the inhibitor treatment was delayed after infection, the inhibitory effect on virus replication also gradually weakened. Especially when the inhibitors were added into cells at 4 and 8 hpi, the suppressive effect on the viral infection was almost abolished. These results indicated that ouabain and PST2238 also inhibited PRRSV-2 infection during the early stage. However, these compounds show no inhibitory effect on viral attachment (Fig. 2F) but still prevented viral internalization (Fig. 2G). Subsequently, the reduction of PRRSV-2 particle internalization was confirmed by laser confocal microscopy. Compared to DMSO-treated cells, most virus particles were retained on the cell membranes but failed to enter the cytoplasm after ouabain and PST2238 treatment (Fig. 2H). In summary, these data indicated that the ATP1A1-mediated signaling pathway is responsible for virus internalization, which can be interfered with treatment of specific chemical ligands.

### ATP1A1 facilitates PRRSV-2 entry via macropinocytosis and caveolae/raft-mediated endocytosis pathways

**Fig 3:**
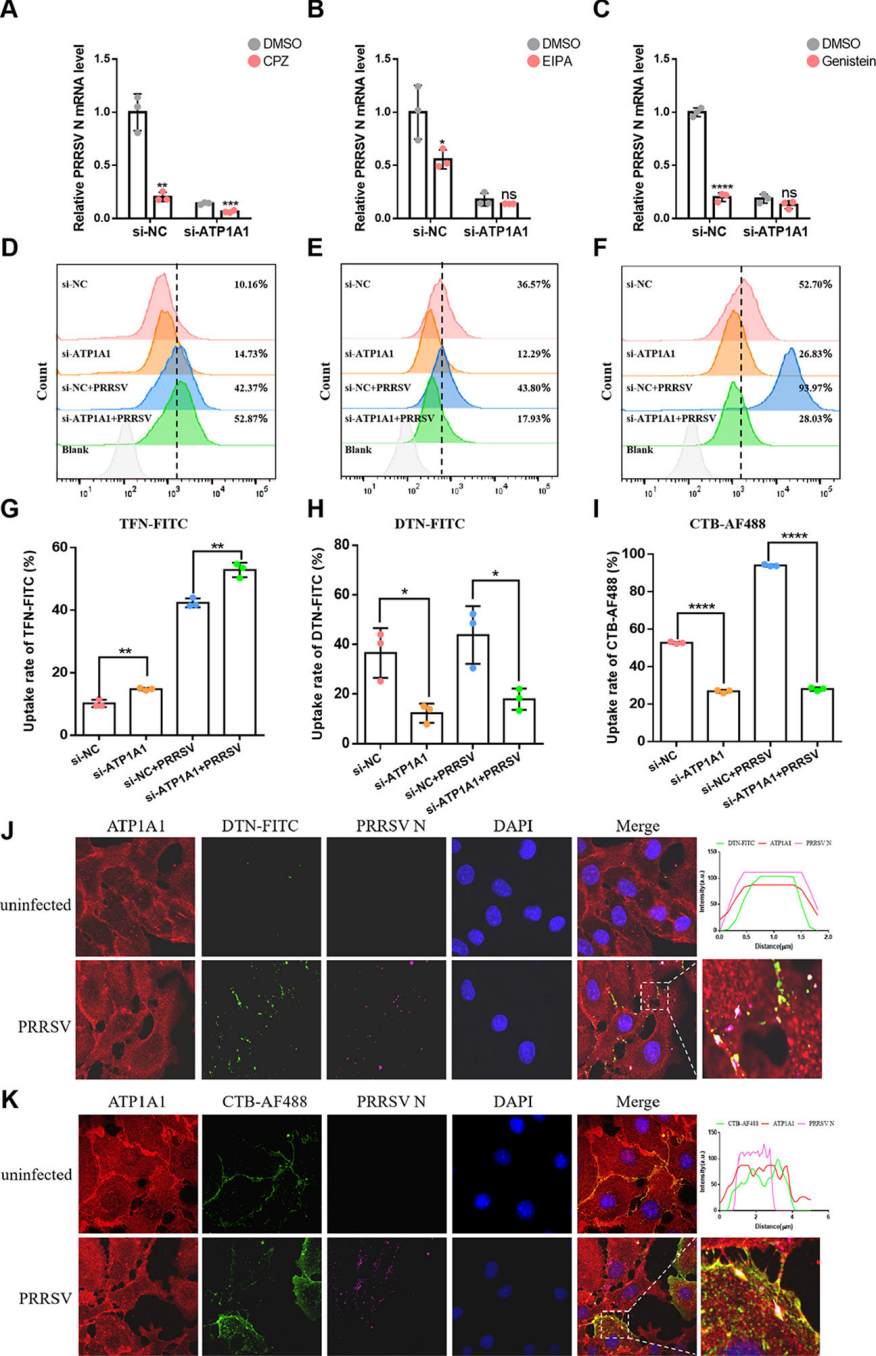
ATP1A1 facilitates PRRSV-2 entry via macropinocytosis and caveolae/raft-mediated endocytosis pathways. (**A–C**) The effect of CPZ (10 µM), EIPA (50 µM), and genistein (50 µM) on PRRSV-2 infection. Marc-145 cells with or without ATP1A1 knockdown were pre-treated with three inhibitors for 2 h, followed by PRRSV HuN4-F112 strain (MOI = 1) infection for another 12 h (with compounds present throughout). Then, the cell lysates were collected to detect viral RNA abundance via qRT-PCR. (**D–I**) Impact of ATP1A1 knockdown on endocytic marker uptake. Marc-145 cells with or without ATP1A1 knockdown were incubated with endocytic markers TFN-FITC (10 µg/mL), DTN-FITC (200 µg/mL), and CTB-AF488 (10 µg/mL) for 1 h. The cells were uninfected or infected with the PRRSV HuN4-F112 strain (MOI = 50). At 1 hpi, cells were collected, and the uptake rates were evaluated by flow cytometry. Representative images for TFN-FITC, DTN-FITC, and CTB-AF488 uptake are shown in panels D, E, and F with quantification provided in panels G, H, and I from three independent replicates. (**J and K**) Colocalization analysis of ATP1A1, PRRSV-2 particles, and DTN-positive macropinosomes or CTB-positive caveolae. Marc-145 cells were incubated with endocytic markers DTN-FITC (200 µg/mL, green) and CTB-AF488 (10 µg/mL, green) for 1 h. Then, the cells were uninfected or infected with the PRRSV HuN4-F112 strain (MOI = 50). At 1 hpi, cells were fixed and stained with rabbit anti-ATP1A1 pAb (red) and mouse anti-PRRSV N mAb (purple). Nuclei were labeled with DAPI (blue). Significant differences were indicated as follows: ns (*P* > 0.05), * (*P* < 0.05), ** (*P* < 0.01), *** (*P* < 0.001), and **** (*P* < 0.0001).

ATP1A1 signaling cascades can result in the induction of endocytosis, including clathrin-mediated (30), caveolae/raft-mediated endocytosis (15), or macropinocytosis (14), which could be involved in PRRSV-2 entry. Hence, the precise endocytic pathway(s) used by PRRSV-2 to enter cells via ATP1A1 were further examined. First, three chemical inhibitors were used, including chlorpromazine (CPZ, inhibitor of the clathrin-mediated endocytosis), ethylisopropyl amiloride (EIPA, inhibitor of the macropinocytosis), and genistein (inhibitor of the caveolae/raft-mediated endocytosis). According to previous studies, CPZ (10 µM), EIPA (50 µM), and genistein (50 µM) did not affect the cell viability of Marc-145 cells (28, 31). Cells with or without ATP1A1 knockdown were pretreated with CPZ, EIPA, or genistein for 2 h, followed by PRRSV-2 infection. As shown in Fig. 3A through C, qRT-PCR showed that the viral RNA levels in all the CPZ-, EIPA-, and genistein-treated cells without ATP1A1 knockdown were significantly decreased, suggesting that PRRSV-2 enters Marc-145 cells via clathrin-, caveolae/raft-mediated endocytosis and macropinocytosis. Notably, PRRSV replication in cells with ATP1A1 knockdown was only further decreased by CPZ treatment, but not by EIPA or genistein treatment, suggesting that ATP1A1 regulates macropinocytosis and caveolae/raft-mediated endocytosis of PRRSV-2.

Subsequently, Marc-145 cells with or without ATP1A1 knockdown were incubated with FITC-labeled Transferrin (TFN-FITC), FITC-labeled Dextran (DTN-FITC), and Alexa Fluor 488-conjugated cholera toxin subunit B (CTB-AF488) to track the internalization process via clathrin-mediated endocytosis, macropinocytosis, and caveolae/raft-mediated endocytosis, respectively. There was a significant decrease in the uptake rates of CTB-AF488 and DTN-FITC in cells with or without PRRSV infection after ATP1A1 knockdown, whereas no reduction of that was found in the TFN-FITC-treated cells, indicating that the endocytic pathways of caveolae/raft-mediated endocytosis and macropinocytosis were inhibited after ATP1A1 knockdown (Fig. 3D through I). Most importantly, ATP1A1 was observed to be co-localized with PRRSV N protein in DTN-positive macropinosomes and CTB-positive caveolae in PRRSV-2 infected Marc-145 cells (Fig. 3J and K). Taken together, these results demonstrated that ATP1A1 facilitates PRRSV-2 entry via macropinocytosis and caveolae/raft-mediated endocytosis pathways.

### ATP1A1-Src signaling cascade-induced phosphorylations of Caveolin-1 and EGFR are required for PRRSV entry

**Fig 4:**
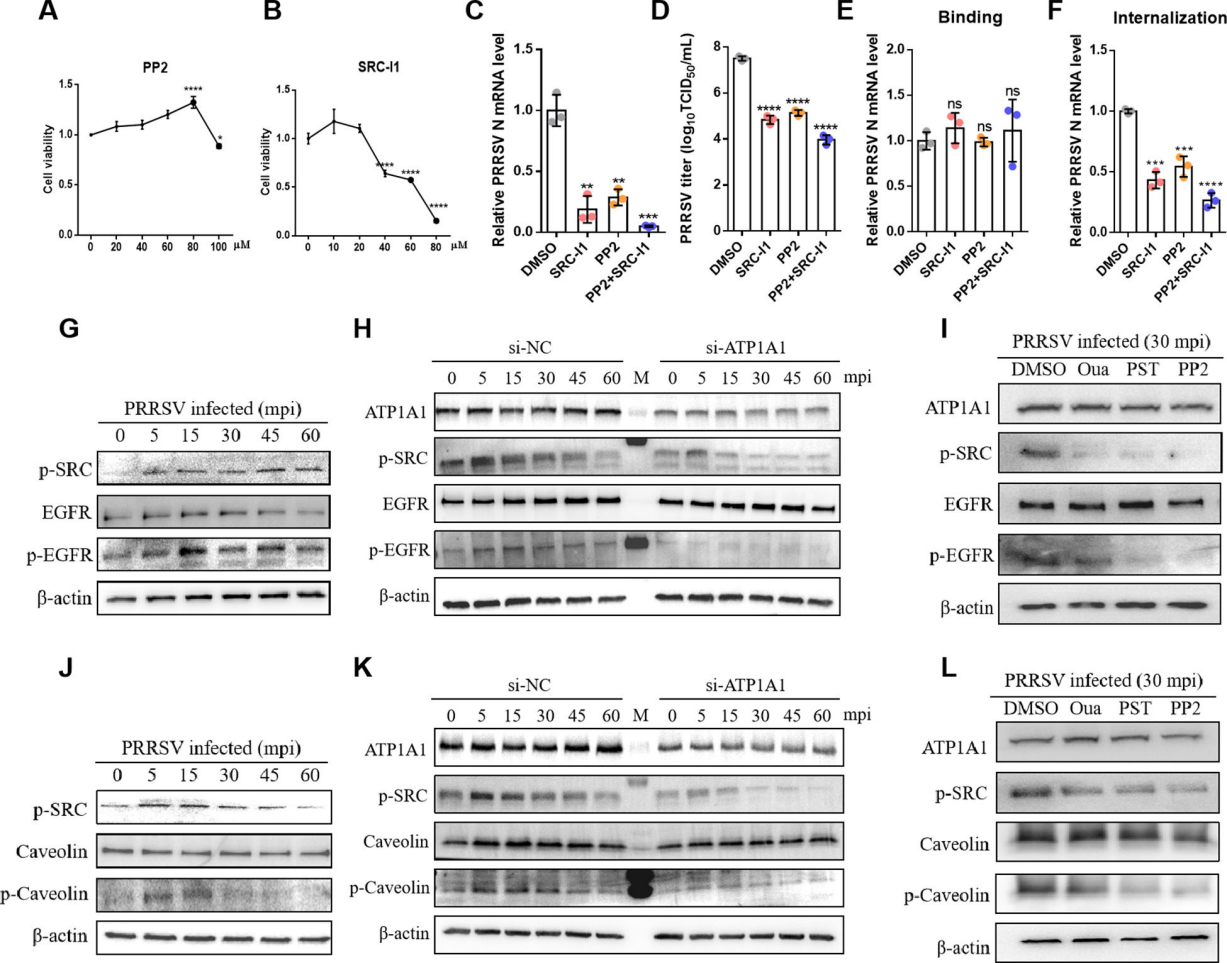
ATP1A1-Src signaling cascade-induced phosphorylations of Caveolin-1 and EGFR are required for PRRSV entry. (**A and B**) Marc-145 cells were treated with 0, 20, 40, 60, 80, and 100 µM PP2 (**A**) and 10, 20, 40, 60, and 80 µM SRCI-1 (**B**) for 48 h. The treated cells were then analyzed using the CCK-8 kit. (**C and D**) The inhibition of SRC-I1 (20 µM) and PP2 (60 µM) on PRRSV infection. Marc-145 cells were pre-treated with two inhibitors for 2 h, followed by PRRSV HuN4-F112 strain (MOI = 1) infection (with compounds present throughout). At 24 hpi, the cell lysates were collected to detect viral RNA abundance via qRT-PCR (**A**), and the supernatants were collected to detect viral titers at 48 hpi (**B**). (**E and F**) The effect of SRC-I1 (10 µM) and PP2 (20 µM) on PRRSV attachment and internalization. Marc-145 cells were pre-treated with two inhibitors for 2 h, then the viral attachment (**E**) and internalization (**F**) assays were conducted in Marc-145 cells. (**G and J**) The activation of the Src-EGFR/caveolin-1 axis upon PRRSV entry. Marc-145 cells were incubated with the PRRSV HuN4-F112 strain (MOI = 50) at 4°C for 1 h. Then, the cells were washed with cold PBS for six times and transferred to 37°C for the indicated time. Cell lysates were analyzed by western blot using p-Src, p-EGFR, EGFR, p-caveolin-1, caveolin-1, and β-actin antibodies. (**H and K**) The effect of ATP1A1 knockdown on Src-EGFR/caveolin-1 axis activation. Marc-145 cells with or without ATP1A1 knockdown were incubated with the PRRSV HuN4-F112 strain (MOI = 50) at 4°C for 1 h. Then the cells were washed with cold PBS for six times and transferred to 37°C for the indicated time. Cell lysates were analyzed by Western blot using p-Src, p-EGFR, EGFR, p-caveolin-1, caveolin-1, and β-actin antibodies. (**I and L**) The effect of ouabain and PST2238 on Src-EGFR/caveolin-1 axis activation. Marc-145 cells were pretreated with ouabain (25 nM) and PST2238 (25 µM) for 2 h, followed by incubating the PRRSV HuN4-F112 strain (MOI = 50) at 4°C for 1 h. The cells were washed with cold PBS for six times and transferred to 37°C for the indicated time. Cell lysates were analyzed by Western blot using p-Src, p-EGFR, EGFR, p-caveolin-1, caveolin-1, and β-actin antibodies. Significant differences were indicated as follows: ns (*P* > 0.05), * (*P* < 0.05), ** (*P* < 0.01), *** (*P* < 0.001), and **** (*P* < 0.0001).

Src kinase is a well-known player in ATP1A1-mediated signaling process and Src phosphorylation mainly correlates to an increase in its kinase activity, leading to ATP1A1 internalization (32). Here, the role of Src kinase activity in PRRSV infection was further investigated. Two Src kinase inhibitors, PP2 and Src-Inhibitor I (Src-I1), were used to conduct subsequent experiments, using concentrations that preliminary studies showed were non-cytotoxic (Fig. 4A and B). As shown in Fig. 4C and D, both Src-I1 and PP2 effectively reduced viral RNA copy number (about 70%–90% and the yield of progeny PRRSV (about 100-fold), and the combination of the two inhibitors exhibited a more powerful antiviral effect. Then, the attachment and internalization assays were conducted in Marc-145 cells pre-treated with two inhibitors. However, these compounds failed to reduce viral attachment (Fig. 4E), but just suppressed viral internalization (Fig. 4F). These results demonstrated that Src kinase activity is important for PRRSV entry.

Epidermal growth factor receptor (EGFR) Tyrosine 845 has been reported to be phosphorylated in response to macropinocytic uptake of RSV, and this process is ATP1A1-Src complex-dependent (14). Thus, we examined the activation of the Src-EGFR axis during PRRSV entry. As shown in Fig. 4G, Src activation was observed as early as 5 min after PRRSV infection. Simultaneously, Y845 phosphorylation of EGFR was activated and peaked at 15 min post-infection (mpi). However, in ATP1A1-knockdown Marc-145 cells, Src and EGFR were not obviously activated compared to the negative control group (Fig. 4H). Similar to ATP1A1 knockdown, ouabain and PST2238 treatment also almost abolished the activation of the Src-EGFR axis at 30 mpi (Fig. 4I), confirming the activation of the Src-EGFR axis during virus entry and is ATP1A1-dependent. Moreover, caveolin-1, the protein marker of caveolae, was found to directly interact with ATP1A1 and was also responsible for caveolae/raft-mediated internalization (33, 34). Hence, the activation of the Src-caveolin-1 axis was further detected during PRRSV entry. As expected, Src-kinase activity also transactivated Caveolin-1 by Y14 phosphorylation as early as 5 mpi, but this effect gradually decreased from 30 mpi (Fig. 4J). Most importantly, both ATP1A1 knockdown and inhibitors treatment interfered with the Src-caveolin-1 axis activation (Fig. 4K and L), indicating that the Src-caveolin-1 axis is also dependent on ATP1A1 during PRRSV entry. In summary, these data suggested that the ATP1A1-Src signaling cascade is required for PRRSV entry by activating EGFR and caveolin-1, leading to initiating macropinocytosis and caveolae/raft-mediated endocytosis.

### ATP1A1 participates in PRRSV infection-induced actin cytoskeleton rearrangement

**Fig 5:**
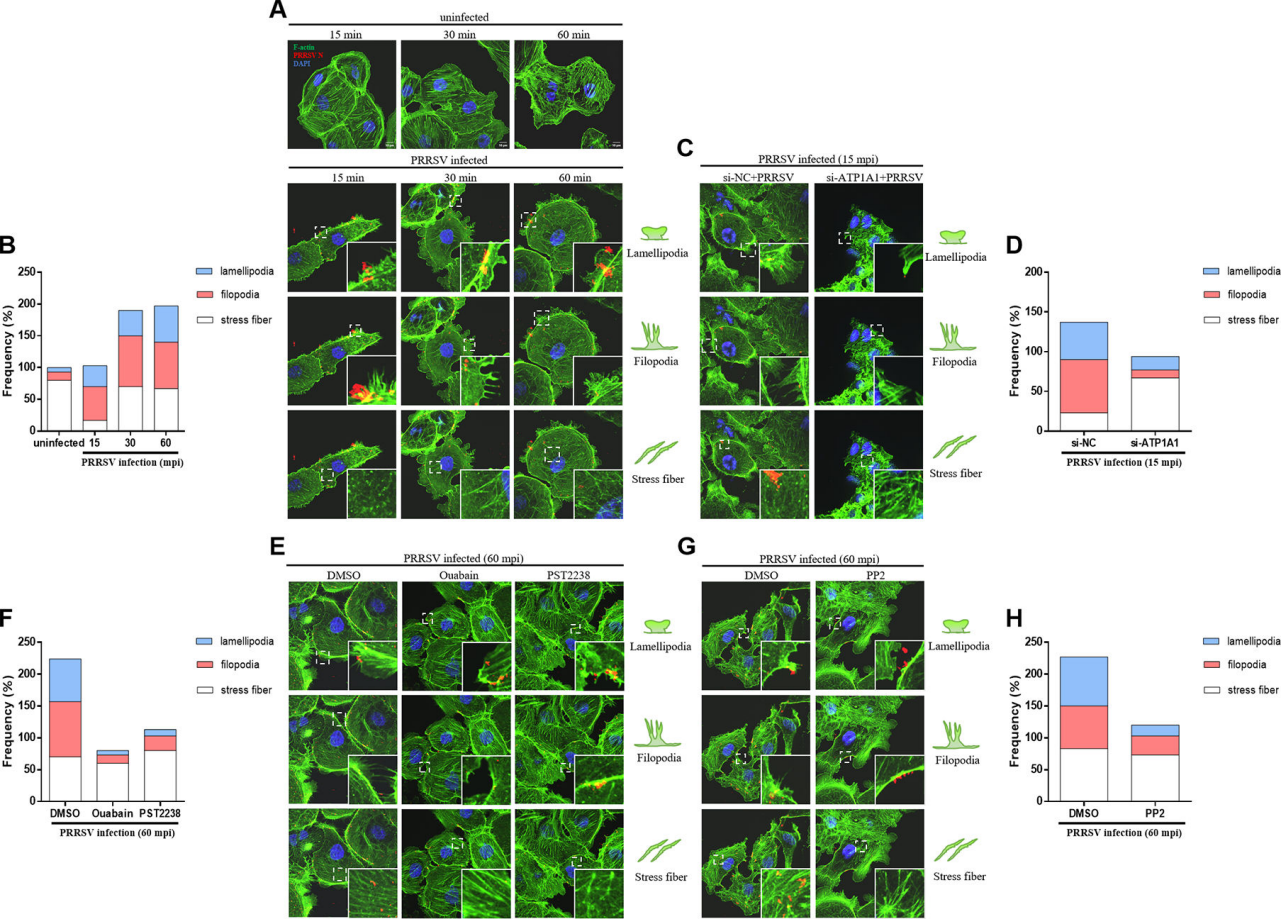
ATP1A1 participates in PRRSV infection-induced actin cytoskeleton rearrangement. Formation of cell protrusions induced by PRRSV infection with or without indicated inhibitor treatment. Cells were stained with Actin-Tracker Green-488 (green), mouse anti-PRRSV N mAb (red), and DAPI (blue) and then examined by laser confocal microscopy. Quantification of the changes in cell ruffles. The cells with F-actin stress fibers, lamellipodia, or filopodia were designated positive cells, and the ratio of the positive cells results was determined from 30 cells from at least three individual fields. (**A and B**) Marc-145 cells were incubated with or without PRRSV HuN4-F112 strain (MOI = 50) at 4°C for 1 h. (**C and D**) The effect of ATP1A1 knockdown on the formation of cell protrusions induced by PRRSV infection at 15 mpi. (**E–H**) The inhibition with ouabain, PST2238, or PP2 on the formation of cell protrusions induced by PRRSV infection at 60 mpi.

The actin cytoskeleton has been implicated in the process of macropinocytosis and caveolae/raft-mediated endocytosis (35, 36). In this study, laser confocal microscopy was carried out to examine whether the dynamic rearrangement of the actin cytoskeleton was induced by PRRSV entry. Marc-145 cells were first incubated with PRRSV at 4°C for 1 h and then incubated at 37°C for the indicated time. As shown in Fig. 5A and B, we observed the dissolution of actin stress fibers and the appearance of filopodia and lamellipodia in PRRSV-infected Marc-145 cells as early as 15 mpi, indicating F-actin polymerization. As PRRSV enters the cells, the stress fibers increased starting from 30 mpi, indicating F-actin depolymerization. Nevertheless, such a phenomenon was not observed in uninfected cells. To further explore whether ATP1A1 participates in actin cytoskeleton rearrangement, the same experiment was conducted in Marc-145 cells with or without ATP1A1 knockdown. Due to the impaired attachment of virions on cells after ATP1A1 knockdown, the sample collection time was moved forward to the initial entry stage (15 mpi) to observe the actin cytoskeleton rearrangement. As expected, in si-NC-transfected cells, the dissolution of actin stress fibers and the appearance of filopodia and lamellipodia were observed. However, in the ATP1A1-knockdown cells with less viral absorption, the arrangement of actin stress fibers was not significantly changed, leading to few filopodia and lamellipodia appearances (Fig. 5C and D). Similarly, when the PRRSV-induced signaling pathway via ATP1A1-Src was inhibited by chemical ligands, including ouabain, PST2238, or PP2, the actin cytoskeleton rearrangement was much less than that in DMSO-treated cells, indicated by few filopodia and lamellipodia appearing in the late entry stage (60 mpi) (Fig. 5E through H). Taken together, these results demonstrated that ATP1A1 participates in PRRSV infection-induced actin cytoskeleton rearrangement, facilitating virus entry.

### PRRSV-2 absorbs to ATP1A1 via the interaction between GP4 C-terminus and ER4

**Fig 6:**
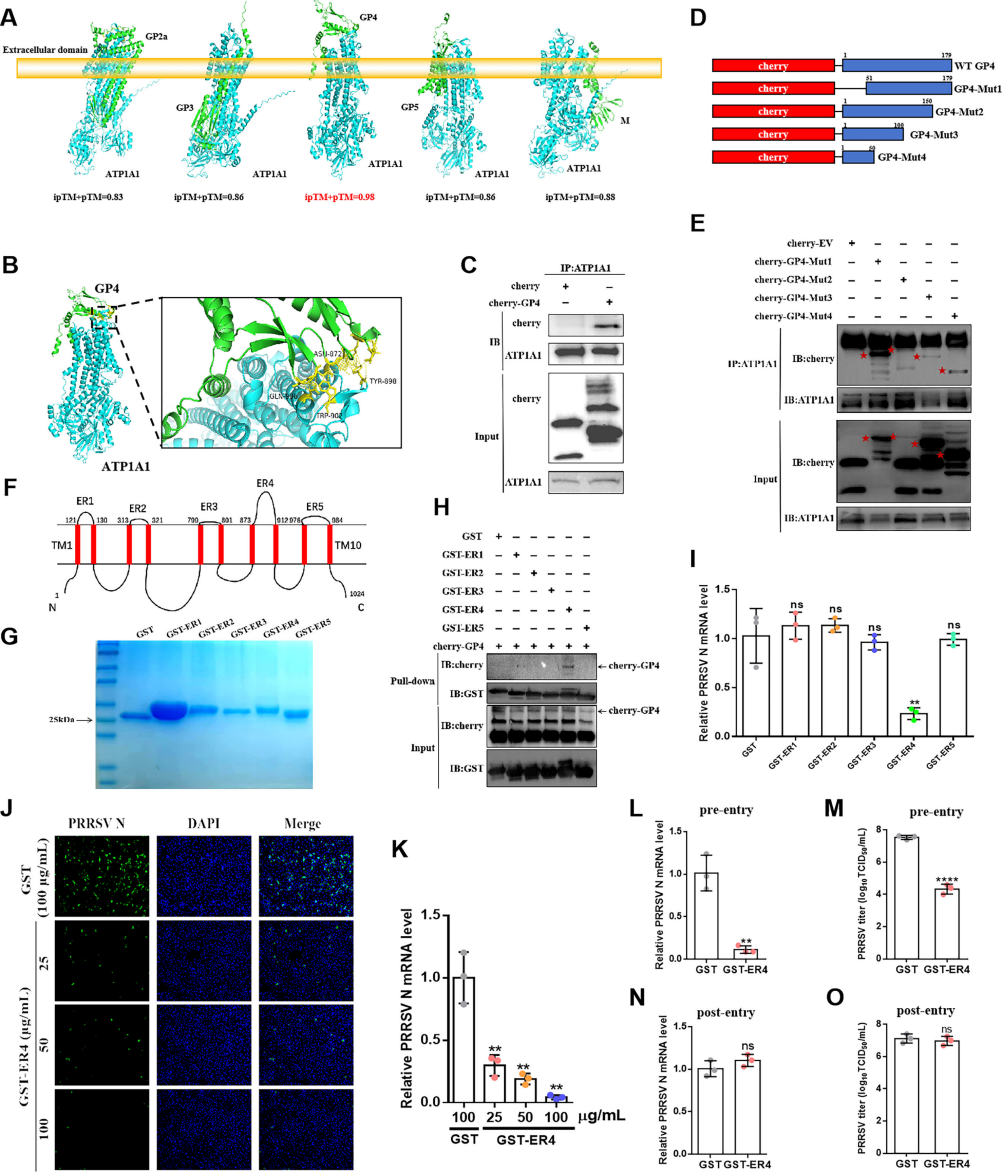
PRRSV-2 binds to ATP1A1 via interaction between GP4 C-terminus and ER4. (**A**) The predicted three-dimensional models of the complex of various PRRSV-2 structural proteins and ATP1A1. (**B**) Molecular docking analysis of ATP1A1 (blue) and GP4 (green). The interacting residues on ATP1A1 are highlighted in black dotted boxes and colored in yellow. (**C**) Analysis of the interaction between ATP1A1 and GP4 via Co-IP assay. Marc-145 cells were transfected with plasmids expressing cherry-GP4, and cells transfected with cherry-EV were used as a negative control. Cell lysates were immunoprecipitated with rabbit anti-ATP1A1 pAb, followed by immunoblotting with rabbit anti-mCherry pAb or rabbit anti-ATP1A1 pAb to reveal GP4 and ATP1A1, respectively. (**D**) Domain structure of four ATP1A1 deletion mutants. (**E**) Domain mapping of GP4 interacting with ATP1A1. Marc-145 cells were transfected with plasmids expressing four GP4 mutants. Cell lysates were immunoprecipitated with rabbit anti-ATP1A1 pAb, followed by immunoblotting with rabbit anti-mCherry pAb or rabbit anti-ATP1A1 pAb to reveal GP4 and ATP1A1, respectively. (**F**) The 2D model illustration of green monkey ATP1A1 was determined from the prediction of transmembrane regions (https://www.novopro.cn/tools/tmhmm.html). (**G**) Purification of ATP1A1 ER proteins. The above five extracellular regions of ATP1A1 were cloned into expression vector pGEX4T and further transferred into *Escherichia coli* BL21 (DE3) strain, followed by IPTG induction. After purification, the purified proteins were subjected to SDS-PAGE analysis. (**H**) Identification of the interaction between GP4 and the extracellular regions of ATP1A1 in GST pulldown. The purified recombinant ATP1A1 ER proteins were coupled to GST beads at 4°C for 2 h, where GST served as control. The beads were incubated with soluble cherry-GP4 protein derived from the supernatant of HEK-293T cells lysate (transient transfection in 6-well plates) at 4°C for overnight. The eluted samples were subjected to IB and detected by anti-mCherry pAb and anti-GST mAb. (**I**) The comparison of ATP1A1-ERs effect on PRRSV-2 replication. Indicated peptides (50 μg/mL) were pre-incubated with PRRSV HuN4-F112 (200 TCID_50_) at 37°C for 2 h. Then, the mixture was added to Marc-145 cells and replaced with DMEM medium containing 2% FBS after 1 h incubation at 37°C. At 24 hpi, virus replication was analyzed by qRT-PCR. (**J and K**) GST-ER4 peptide blocks PRRSV-2 infection in a dose-dependent manner. The GST-ER4 peptide in various concentrations was incubated with PRRSV HuN4-F112 (200 TCID_50_) at 37°C for 2 h. Then, the mixture was added to Marc-145 cells and replaced with DMEM medium containing 2% FBS after 2 h incubation. At 24 hpi, virus replication was identified in IFA (**J**) and qRT-PCR (**K**). (**L and M**) The pre-entry assay. The GST-ER4 peptide (100 μg/mL) was incubated with PRRSV HuN4-F112 (200 TCID_50_) at 37°C for 2 h. Then, the mixture was added to Marc-145 cells and replaced with DMEM medium containing 2% FBS after 2 h incubation. At 24 hpi, virus replication was detected by qRT-PCR (**L**), and viral titers were determined at 48 hpi (**M**). (**N and O**) The post-entry assay. Marc-145 cells were infected with PRRSV HuN4-F112 (200 TCID_50_) at 37°C for 2 h. Then, the cells were inoculated by GST-ER4 peptide (100 μg/mL), followed by washing three times with PBS after 2 h incubation. After this, the peptide-containing medium was replaced with DMEM containing 2% FBS. At 24 hpi, virus replication was detected by qRT-PCR (**N**), and viral titers were determined by detecting TCID_50_ at 48 hpi (**O**). Significant differences were indicated as follows: ns (*P* > 0.05), * (*P* < 0.05), ** (*P* < 0.01), *** (*P* < 0.001), and **** (*P* < 0.0001).

The first step of PRRSV-2 infection of the host cells is a receptor-mediated process dependent on various viral structural proteins, including GP2, GP3, GP4, GP5, or M (18). To find out which viral component is responsible for ATP1A1-mediated viral internalization, the complex structures of ATP1A1 with these above entry-associated proteins were predicted. As shown in Fig. 6A, among all of the predicted models, GP4-ATP1A1 complex received the highest score of iPTM (interface predicted alignment score) and pTM (predicted alignment score), indicating the more reliable protein–protein interaction. Moreover, GP4, recognized by the extracellular domain of ATP1A1, also exhibited a classical ligand-receptor binding pattern. Molecular docking analysis suggested the C-terminus of GP4 interacted with the residues Asn872, Tyr898, Trp902, and Gln906 of ATP1A1 via hydrogen bonds (Fig. 6B). Furthermore, the interaction between GP4 and endogenous ATP1A1 was confirmed by a Co-IP assay (Fig. 6C). To determine the critical domain of GP4 for interacting with ATP1A1, GP4 protein was orderly truncated to perform domain mapping (Fig. 6D). Co-IP assay revealed that among four mutants, GP4-Mut1, which lacks the N-terminal 50 amino acids (AA), exhibited the strongest ability to interact with ATP1A1. However, once the C-terminal 30AA is lacking, the binding capacity of GP4-Mut2/3/4 to ATP1A1 was significantly impaired (Fig. 6E). ATP1A1 is a 10-transmembrane protein, consisting of five extracellular regions (ER1–ER5) and 10 transmembrane regions (TTM1–TM10) (Fig. 6F). Due to the complex structure, it is difficult to express and purify the structurally complete and correctly folded ATP1A1 protein. Thus, five glutathione S-transferase (GST)-labeled synthetic peptides of the extracellular regions of ATP1A1 (designated GST-ER1/2/3/4/5) were generated and purified first. As shown in Fig. 6G, in SDS-PAGE analysis, the expressions of GST-ER1 to GST-ER5 were identified at the molecular weight of about 25–30 kD, and the GST-ER proteins were used in a GST pulldown assay with soluble cherry-GP4 protein generated from HEK-293T cells by transient transfection. The results shown only GST-ER4 successfully pulled the cherry-GP4 protein down (Fig. 6H). These data determined PRRSV-2 GP4 binds to ATP1A1-ER4 mainly via its C-terminus.

To further determine whether the interaction between ATP1A1 and GP4 would influence PRRSV-2 entry, peptide-blocking assays were performed. PRRSV-2 virions were pre-inoculated with GST-ER1/2/3/4/5 or GST control peptides (50 μg/mL), respectively, at 37°C for 2 h prior to the infection of Marc-145 cells. As shown in Fig. 6I, only GST-ER4 peptide showed significant inhibition on PRRSV-2 replication. Then, the effect of GST-ER4 against PRRSV-2 was tested in a dose gradient (25, 50, and 100 µg/mL) evaluation. The results of qRT-PCR and IFA showed a significant reduction in PRRSV N transcription levels and protein expression under the treatment with GST-ER4 peptide in a dose-dependent manner, compared with the GST protein treatment cells (Fig. 6J and K). Subsequently, the following experiments were performed: (i) ppre-entry assay, in which the GST-ER4 peptide was co-incubated with PRRSV-2 prior to viral entry, and (ii) post-entry assay, in which the GST-ER4 peptide was added into cells after viral internalization. The results showed that GST-ER4 peptide exhibited a significant inhibitory effect on both viral gene copy number and viral titers exclusively in the ppre-entry assay (Fig. 6L and M), but no inhibition on viral replication was observed in the post-entry assay (Fig. 6N and O). Taken together, these results demonstrate that synthetic ATP1A1-ER4 peptide effectively blocks PRRSV-2 infection by disrupting the viral entry process.

### PRRSV-2 particles are transported to ATP1A1/CD163-containing early endosomes

**Fig 7:**
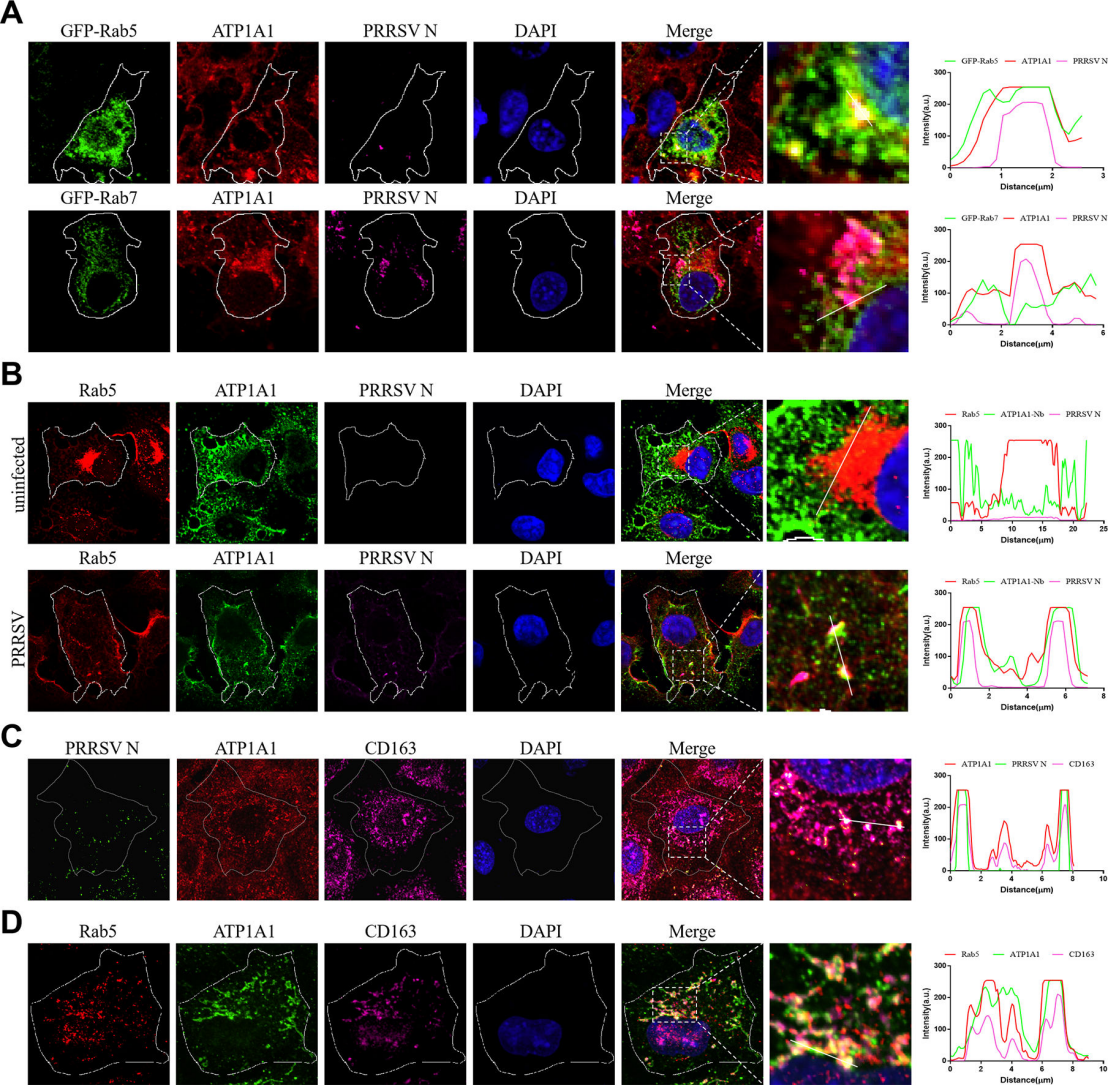
PRRSV-2 particles are transported to ATP1A1-/CD163-containing early endosomes. Laser confocal microscopy was carried out in these experiments. (**A**) Colocalization analysis of ATP1A1, PRRSV-2 particles, and GFP-Rab5-positive early endosomes or GFP-Rab7-positive late endosomes. Marc-145 cells were transfected with the plasmids overexpressing GFP-Rab5 or GFP-Rab7. After 24 h transfection, cells were infected with the PRRSV HuN4-F112 strain (MOI = 50). At 1 hpi, cells were fixed and stained to observe ATP1A1 (red), GFP-Rab5 (green), and PRRSV N (purple). Nuclei were labeled with DAPI (blue). At 2 hpi, cells were fixed and stained to observe ATP1A1 (red), GFP-Rab7 (green), and PRRSV N (purple). (**B**) Marc-145 cells were infected with the PRRSV HuN4-F112 strain (MOI = 50) or mock-infected. At 1 hpi, cells were fixed and stained with rabbit anti-Rab5 pAb (red) and mouse anti-PRRSV N mAb (purple) first. Then, the endogenous ATP1A1 was stained with GFP-ATP1A1-Nb (green), and nuclei were labeled with DAPI (blue). (**C**) Marc-145 cells were infected with the PRRSV HuN4-F112 strain (MOI = 50). At 1 hpi, cells were fixed and stained with rabbit anti-ATP1A1 pAb (red) and mouse anti-CD163 mAb (purple) first. Then, PRRSV N was stained with FITC-labeled anti-PRRSV N mAb (green), and nuclei were labeled with DAPI (blue). (**D**) Marc-145 cells were infected with the PRRSV HuN4-F112 strain (MOI = 50). At 1 hpi, cells were fixed and stained with rabbit anti-Rab5 pAb (red) and mouse anti-CD163 mAb (purple) first. Then, the endogenous ATP1A1 was stained with GFP-ATP1A1-Nb (green), and nuclei were labeled with DAPI (blue).

PRRSV-2 has been reported to transport in early endosomes but not in late endosomes after internalization (37). To investigate whether ATP1A1 enters into early endosomes together with PRRSV-2 particles, two fluorescent plasmids, GFP-Rab5 (served as the marker protein of early endosomes) and GFP-Rab7 (served as the marker protein of late endosomes), were subsequently constructed. As shown in Fig. 7A, the colocalization of ATP1A1 with virions and GFP-Rab5 was observed in Marc-145 cells with PRRSV-2 infection for 1 h. However, GFP-Rab7 rarely contained any ATP1A1 clusters with viral particles in cells infected by PRRSV-2 for 2 h. In order to find out whether ATP1A1 and virions colocalize in endogenous Rab5-positive early endosomes, a nanobody that specifically recognizes ATP1A1 ER4 (ATP1A1-Nb) was screened as described in the previous study (38). Furthermore, GFP-labeled ATP1A1-Nb was produced and used to label ATP1A1 in subsequent studies. Consistently, ATP1A1 and virions were co-transported to endogenous early endosomes at 1 hpi (Fig. 7B). These data demonstrated ATP1A1 enters into early endosomes along with PRRSV-2 after internalization.

CD163, as the pivotal functional receptor for PRRSV-2, is known to co-internalize with virions into early endosomes and mediate viral uncoating (23). Hence, we further investigated whether there is any cooperation between ATP1A1 and CD163 during this process. The confocal image results showed that ATP1A1 colocalizes with CD163 and virions (Fig. 7C). Simultaneously, as shown in Fig. 7D, the colocalization of ATP1A1 with CD163 and Rab5 was also observed. Collectively, PRRSV-2 particles transport to ATP1A1/CD163-containing early endosomes after internalization.

### ATP1A1 recruits PRRSV-2 particles for further CD163-mediated viral entry step

**Fig 8:**
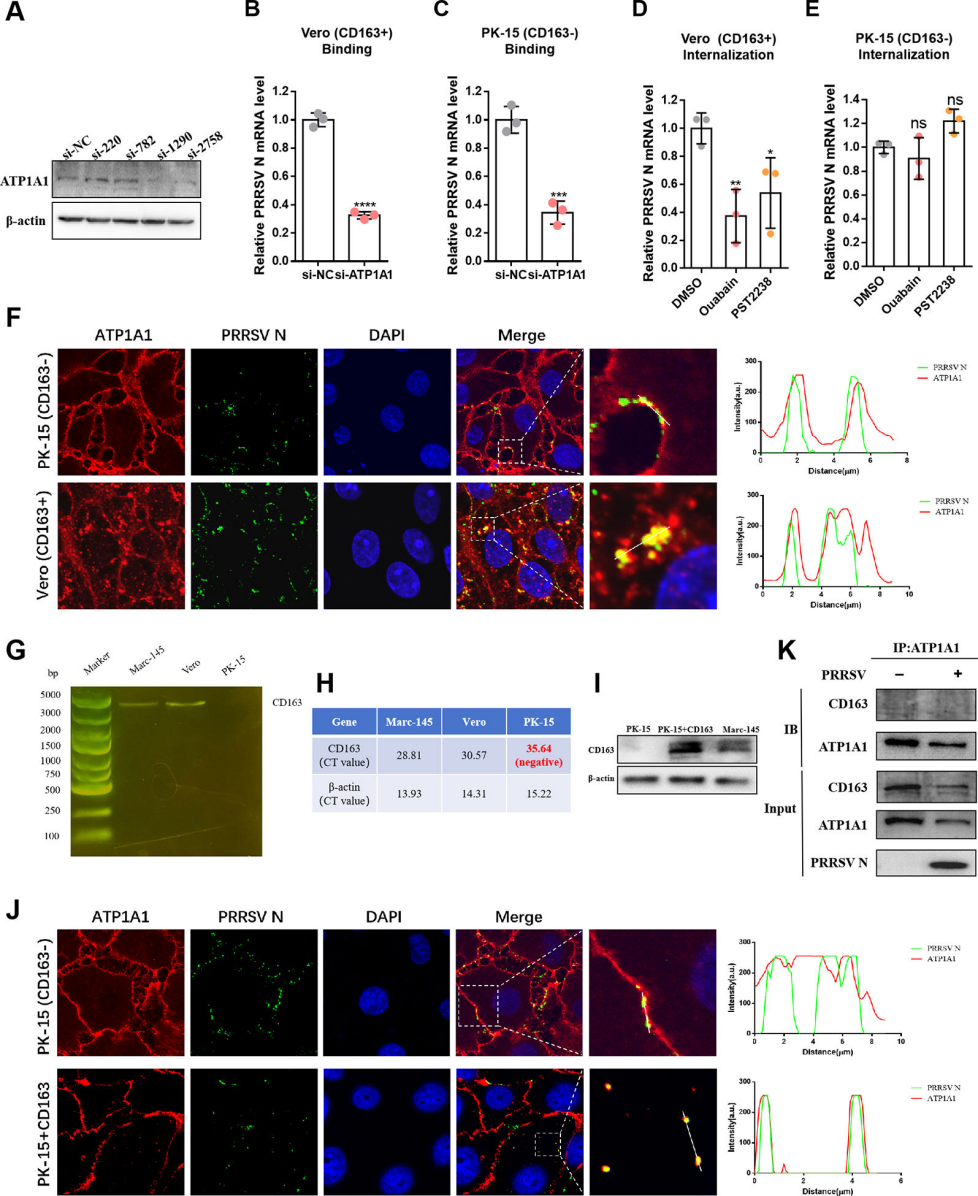
ATP1A1 recruits PRRSV-2 particles for further CD163-mediated viral entry step. (**A**) Four siRNAs for the knockdown of Sus scrofa ATP1A1 were constructed. All of them were transfected into PK-15 cells, and si-NC was used as a negative control. The cell lysates were collected to detect the knockdown efficiency via western blotting analysis. (**B and C**) ATP1A1 knockdown significantly reduced PRRSV-2 attachment in Vero and PK-15 cells. Cells were transfected with siRNA targeting ATP1A1 or negative control for 48 h. For the viral attachment assay, PRRSV HuN4-F112 strain (MOI = 50) infected cells at 4°C for 2 h and washed with cold PBS six times. Cell lysates were collected to detect viral RNA abundance via qRT-PCR. (**D and E**) The effect of ouabain (25 nM) and PST2238 (25 µM) on PRRSV-2 internalization in Vero and PK-15 cells. Cells were pre-treated with two inhibitors for 2 h, followed by PRRSV-2 infection. For viral internalization assay, PRRSV HuN4-F112 strain (MOI = 50) infected cells at 4°C for 2 h. Then, the cells were washed with cold PBS for six times and transferred to 37°C for 2 h incubation to complete virus internalization. The cells were washed six times with acidic PBS (pH = 2.0) to remove the uninternalized virus particles. After that, the cell lysates were collected to detect viral RNA abundance via qRT-PCR. (**F**) Confocal microscopy analysis of PRRSV-2 and ATP1A1 in Vero and PK-15 cells. Cells were infected with the PRRSV HuN4-F112 strain (MOI = 50) at 4°C for 2 h. Then, the cells were washed with cold PBS for six times and transferred to 37°C for 2 h incubation to complete virus internalization. After that, cells were fixed and stained with rabbit anti-ATP1A1 pAb (red) and mouse anti-PRRSV N mAb (green). Nuclei were labeled with DAPI (blue). (**G and H**) PCR (**G**) and qPCR (**H**) analysis of CD163 molecule exists in Vero cells. Marc-145 cells were used as the positive control, and PK-15 cells were used as the negative control. (**I**) Detection of CD163 expression level in PK-15 cells transfected with CD163 overexpressing plasmid. (**J**) Confocal microscopy analysis of PRRSV-2 and ATP1A1 in PK-15 cells with or without CD163 overexpression. PK-15 cells were transfected with the plasmid overexpressing CD163 or the empty control plasmid. After 48 h transfection, cells were infected with the PRRSV HuN4-F112 strain (MOI = 50) at 4°C for 2 h. Then, the cells were washed with cold PBS for six times and transferred to 37°C for 2 h incubation to complete virus internalization. After that, cells were fixed and stained with rabbit anti-ATP1A1 pAb (red) and mouse anti-PRRSV N mAb (green). Nuclei were labeled with DAPI (blue). (**K**) Detection of the interaction between ATP1A1 and CD163 via Co-IP assay. Marc-145 cells were transfected or mock infected with the PRRSV HuN4-F112 strain (MOI = 10) for 2 hpi. Cell lysates were immunoprecipitated with rabbit anti-ATP1A1 pAb, followed by immunoblotting with mouse anti-CD163 mAb and mouse anti-PRRSV N mAb to reveal ATP1A1, CD163, PRRSV N, respectively. Significant differences were indicated as follows: ns (*P* > 0.05), * (*P* < 0.05), ** (*P* < 0.01), *** (*P* < 0.001), and **** (*P* < 0.0001).

For non-susceptible cell lines, such as PK-15 cells, PRRSV-2 can absorb to the cell surface but cannot internalize into cytoplasm (24). In contrast, for another non-susceptible Vero cells, PRRSV-2 has been reported to realize both attachment and internalization, yet still fails to establish a productive infection (25). The underlying mechanism for this phenomenon has not been clearly elucidated. ATP1A1 is widely expressed in mammalian cells, and there is high homology between ATP1A1 genes of porcine and green monkey. Theoretically, ATP1A1 could mediate the attachment and internalization of PRRSV-2 in non-susceptible cells. Therefore, the verification experiments were conducted in PK-15 and Vero cells. First, the ATP1A1 knockdown effects of four ATP1A1 siRNAs were detected in PK-15 cells (Fig. 8A). As expected, compared to cells transfected with si-NC, the attachment of PRRSV-2 was significantly reduced in both ATP1A1-knockdown Vero and PK-15 cells (Fig. 8B and C). However, when the cells were treated with the ATP1A1 chemical ligands ouabain and PST2238, the reduction in viral internalization was observed only in Vero cells, with no similar effect seen in PK-15 cells (Fig. 8D and E). Interestingly, further laser confocal microscopy revealed that in Vero cells, ATP1A1 and viral particles were clearly internalized and co-localized in the cytoplasm. In contrast, in PK-15 cells, they only co-localized on the cell surface (Fig. 8F). This discrepancy suggests the involvement of other molecule(s) in the following process of PRRSV-2 entry.

Notably, the same as Marc-145 cells, Vero cells are also derived from African green monkey kidney, which naturally express CD163 molecule (39), consistent with our results (Fig. 8G and H). As CD163 is the key receptor for PRRSV-2 entry, we hypothesized that the co-internalization of ATP1A1 with PRRSV-2 is also associated with CD163. Consequently, laser confocal microscopy was performed on PK-15 cells with or without overexpressing CD163, following with the CD163 expression level that has been checked in advance (Fig. 8I). The results showed that, in contrast to original PK-15 cells, ATP1A1 and PRRSV-2 virions co-localized within the cytoplasm of CD163-overexpressing PK-15 cells (Fig. 8J). However, no direct interaction between ATP1A1 and CD163 was detected in Marc-145 cells regardless of the presence or absence of PRRSV-2 infection by Co-IP assay (Fig. 8K). Together, these findings indicate that the co-internalization of ATP1A1 with PRRSV-2 is a CD163-dependent process.

### ATP1A1-ER4 blocker provides broad inhibition against various PRRSV-2 lineages in PAMs and Marc-145 cells

**Fig 9:**
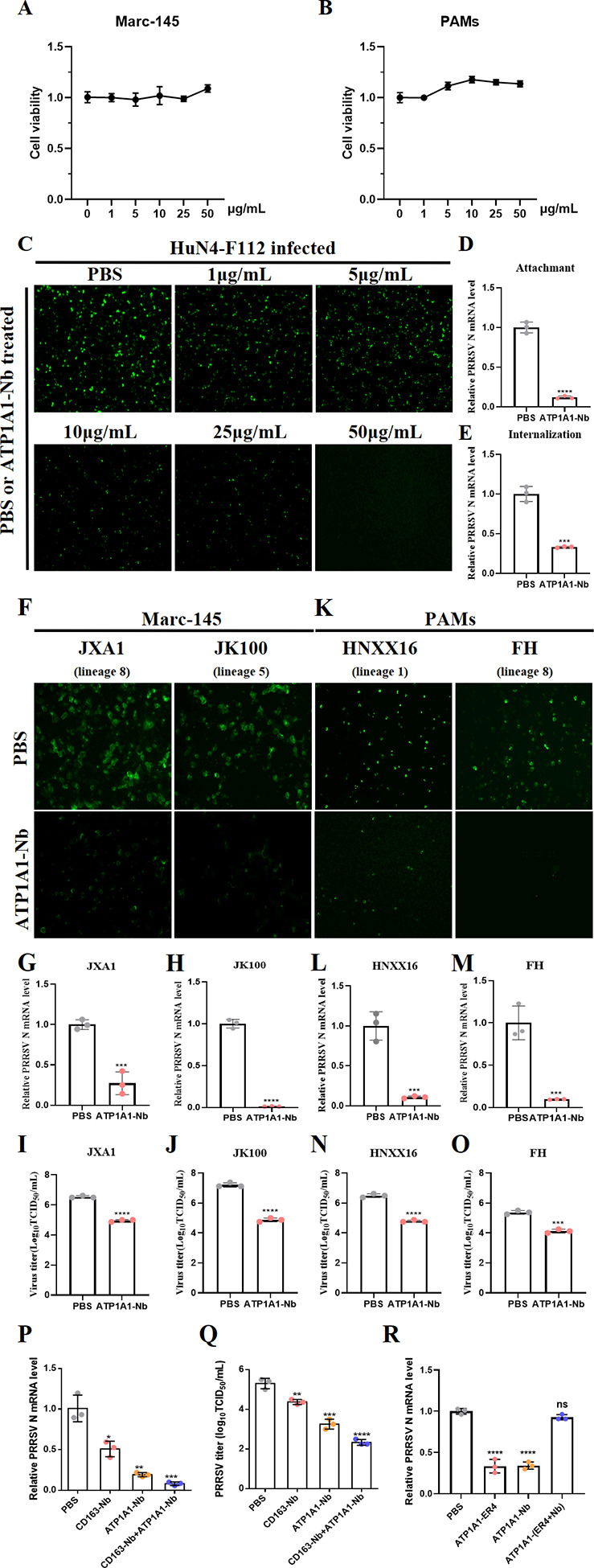
ATP1A1-ER4 blocker provides broad inhibition against various PRRSV-2 lineages in PAMs and Marc-145 cells. (**A and B**) The cytotoxicity of the nanobody targeting with ATP1A1 on Marc-145 cells and PAMs. Marc-145 cells (**A**) and PAMs (**B**) were treated with 0, 1, 5, 10, 25, and 50 µg/mL ATP1A1-Nb for 48 h. The treated cells were then analyzed using the CCK-8 kit. (**C**) Marc-145 cells were incubated with ATP1A1-Nb at indicated concentrations (50, 25, 10, 5, and 1 μg/mL) at 37°C for 2 h, followed by PRRSV HuN4-F112 (200 TCID50) infection. Then, the cells were fixed at 48 hpi and stained for PRRSV N protein (green). (**D and E**) The effect of ATP1A1-Nb on PRRSV-2 attachment and internalization. For the viral binding assay, Marc-145 cells were pre-incubated with ATP1A1-Nb (50 µg/mL) at 37°C for 2 h, followed by PRRSV HuN4-F112 infection. Then, the viral attachment (**D**) and internalization (**E**) assays were conducted. (**F–J**) Antiviral activity of ATP1A1-Nb against PRRSV-2 JXA1 (Lineage 8) and JK100 (Lineage 5) (200 TCID50) in Marc-145 cells. IFA detection of PRRSV N protein for 48 hpi (**F**); qRT-PCR quantification of viral gene copy numbers for 24 hpi (**G and H**); virus titers determined by TCID₅₀ assay for 48 hpi (**I and J**). (**K–O**) Antiviral efficacy of ATP1A1-Nb in PAMs infected with PRRSV-2 HNXX16 (Lineage 1) or FH (Lineage 8) (200 TCID50). IFA for 48 hpi (**K**); qRT-PCR analysis of viral gene copy numbers for 24 hpi (**L and M**) and virus titers detection for 48 hpi (**N and O**). (**P and Q**) Evaluation of the effects of ATP1A1-Nb and CD163-Nb on PRRSV-2. PAMs were treated with different Nbs (50 μg/mL) or CD163-Nb and ATP1A1-Nb were mixed at a ratio of 50:50 (wt/wt) at 37°C for 2 h, followed by PRRSV-2 HNXX16 strain (200 TCID50) infection. qRT-PCR analysis of viral gene copy numbers for 24 hpi (**P**), and virus titers detection for 48 hpi (**Q**). (**R**) Evaluation of the effects of pre-incubation of GST-ER4 with ATP1A1-Nb on PRRSV-2. The GST-ER4 peptide (50 μg/mL) and ATP1A1-Nb were pre-incubated alone or both together at a ratio of 50:50 (w/w) at 37°C for 2 h, followed by PRRSV-2 HNXX16 strain (200 TCID50) incubation for another 2 h. Then, the mixture was added to PAMs and replaced with DMEM medium containing 2% FBS after 2 h incubation. qRT-PCR analysis of viral gene copy numbers for 24 hpi. Significant differences were indicated as follows: ns (*P* > 0.05), * (*P* < 0.05), ** (*P* < 0.01), *** (*P* < 0.001), and **** (*P* < 0.0001).

Given the critical role of ATP1A1-ER4 in PRRSV-2 invasion, antiviral strategies could be developed by targeting ER4. As a special ER4 blocker, the anti-PRRSV-2 activity of our ATP1A1-Nb used above was further explored. Within a non-cytotoxic range (Fig. 9A and B), ATP1A1-Nb suppressed replication of the PRRSV HuN4-F112 strain in a clear dose-dependent manner (Fig. 9C). To elucidate the detailed antiviral mechanism, the effect of ATP1A1-Nb on viral attachment and internalization was examined. As expected, ATP1A1-Nb treatment markedly reduced both PRRSV-2 virions absorption and internalization (Fig. 9D and E), indicating that ATP1A1-Nb interferes with early steps of viral entry via blocking ATP1A1 ER4, consistent with our initial hypothesis. PRRSV-2 has been characterized as various lineages (40). Subsequently, we evaluated the antiviral efficacy of ATP1A1-Nb against PRRSV-2 JXA1 (Lineage 8) and JK100 (Lineage 5) strains in Marc-145 cells. IFA revealed a pronounced reduction in PRRSV N protein expression (Fig. 9F). Also, quantification by qRT-PCR (Fig. 9G and H) and determination of infectious virus titers (Fig. 9I and 9J) confirmed a significant decrease in viral replication after ATP1A1-Nb treatment.

PAMs are the primary targets of PRRSV-2 *in vivo*; it is necessary to explore whether ATP1A1-Nb has the same inhibitory effect in PAMs. Hence, the inhibitory activity of ATP1A1-Nb against HNXX16 (NADC30-like strain, Linage 1) and FH (Linage 8) strains was further assessed. Consistent with the results obtained in Marc-145 cells, ATP1A1-Nb treatment substantially reduced PRRSV N protein signals (Fig. 9K), diminished viral gene copy numbers (Fig. 9L and M), and lowered infectious titers (Fig. 9N and O). Finally, to further investigate the potential synergistic effect of ATP1A1 and CD163 on PRRSV-2 infection, a CD163-specific nanobody (CD163-Nb) also with virus-blocking activity and ATP1A1-Nb were applied either individually or in combination to analyze their inhibitory effects. As shown in Fig. 9P and Q, the combination of two nanobodies led to a stronger inhibition on both viral genome copy number and viral titers in PAMs, compared to either CD163-Nb or ATP1A1-Nb in solo at the same amount. Finally, to further demonstrate the antiviral effect of ATP1A1-Nb is specifically dependent on ATP1A1 ER4-PRRSV interaction, an experiment was performed to demonstrate whether pre-incubation with ATP1A1-Nb could neutralize one another. Indeed, we found that pre-incubation with either GST-ER4 or ATP1A1-Nb alone reduced infectivity, but pre-incubation with both together showed no antiviral effect (Fig. 9R). Taken together, these results demonstrated ATP1A1-Nb targeting with ATP1A1 ER4 can exert robust antiviral activity across multiple PRRSV-2 lineages in both continuous and primary cells, and it has a synergistic effect with the CD163-blocking strategy.

## DISCUSSION

To successfully establish infection, viruses must first gain access to the intracellular environment of the host cells. This critical step depends on the presence of specific surface factors that mediate viral attachment and internalization. In the case of PRRSV-2, the precise mechanisms underlying receptor-mediated entry remain incompletely understood. Here, ATP1A1, the major subunit of Na^+^-K^+^-ATPase (NKA), is identified to promote both PRRSV-2 attachment and internalization. Silencing of ATP1A1 markedly reduced PRRSV-2 attachment in Marc-145 cells, leading to a significant decrease in viral RNA copies and progeny titers. Simultaneously, the capacity of PRRSV-2 binding to non-susceptible cells with ATP1A1 knockdown, such as PK-15 and Vero cells, is also significantly decreased. These data indicate that ATP1A1 facilitates PRRSV-2 binding on various types of cells. It should be noted that ATP1A1 participates in viral recognition in a virus-specific manner but not a general mechanism used by all of the viruses. For instance, it has been proved that influenza A virus (IAV) infection was not affected by ATP1A1 knockdown (12). Although ATP1A1 is also responsible for the attachment of PEDV (13); however, in other viruses, such as WSSV, MHV, and FIPV, ATP1A1 is not responsible for virus binding but only participates in the subsequent viral internalization (12, 15). Following virus binding, entry factors usually cluster and result in association with lipid domains and activation of signaling pathways (5). For instance, herpes simplex virus attaches to cell surface glycosaminoglycans and induces lipid raft clustering, increasing the incorporation and oligomerization of CXCR4 receptors into these microdomains (41). Similarly, in live cells, Rift Valley fever and Ukuniemi viruses-induced clustering of cell surface DC-SIGN can be visualized (42). In our study, we also observed the phenomenon in which ATP1A1 rapidly forms clusters in the plasma membrane following infection with PRRSV, suggesting the activation of a signaling pathway that triggers endocytic uptake of the entry factor cluster and the ligand.

Ouabain, the classical chemical ligand of NKA, at high concentrations (more than hundreds of nM), can inhibit the ion pump function of the NKA enzyme, leading to changes in intracellular sodium, potassium, and calcium concentrations. While at low concentrations, it just triggers signal transduction pathways via ATP1A1 without affecting the ion pump function (43). PST2238 is a competitive inhibitor of ouabain that shares a common binding site on the extracellular domain of ATP1A1 and inhibits ouabain binding and signaling (14). PRRSV-2 infection can be significantly reduced by these chemical ligands treatment via preventing viral internalization but not viral attachment, due to their only regulating the signaling pathway without affecting ATP1A1 protein expression level according to our confocal images and western blotting analysis. Mechanistically, ouabain has been reported to activate the ATP1A1-related signaling cascade. Hence, the antiviral mechanism of ouabain may involve competing and inhibiting the signaling pathways induced by PRRSV-2 internalization. The antiviral mechanism of PST2238 seems straightforward; that is, PST2238 directly blocks the ATP1A1-related signaling cascade required for PRRSV-2 entry because it does not induce any signaling activation by itself. Taken together, these data demonstrate that ATP1A1 not only facilitates PRRSV attachment but also participates in the internalization process as an important signaling transducer.

The function of ATP1A1 as a signaling transducer is well-established that it interacts with Src kinase and initiates downstream cascades necessary for endocytosis (44). Src kinase is a membrane-associated non-receptor tyrosine kinase, playing a vital role in the signal transduction pathways provoked by many extracellular stimuli such as growth factors and ligands of G protein-coupled receptors (45). Upon stimulation, ATP1A1/Src receptor complex leads to the tyrosine phosphorylation and activation of the associated Src, recruitment of additional Src, and the initiation of the signal transduction processes (44). Here, similar to a previous study (28), pharmacological inhibition of Src using PP2 or Src-I1 suppressed PRRSV-2 entry, confirming that ATP1A1-Src signaling is important for PRRSV-2 uptake. Furthermore, ATP1A1-Src signaling mainly depends on the cholesterol-rich microdomains called caveolae because NKA and Src co-enrich in caveolar fraction, where EGFR acts as a key partner (44, 46). In this study, upon PRRSV-2 exposure, ATP1A1 triggers the activation of Src kinase, leading to the phosphorylation of EGFR at Tyr845 and caveolin-1 at Tyr14 as early as 5 mpi, which are two pivotal events known to induce macropinocytosis and caveolae/raft-mediated endocytosis (14, 34). Notably, the activation of the related signaling pathways is dynamically regulated, indicating a clear activation starting at 5 mpi but a gradual inactivation after 30 mpi. This phenomenon may be due to that PRRSV-2 recruitment and trafficking require rapid F-actin polymerization and depolymerization events, similar to the results reported previously (12) and in this study. Simultaneously, chemical inhibition assays further substantiated ATP1A1-mediated endocytic pathways induced by PRRSV-2. Similar to previous studies (28, 31), treatment with CPZ, genistein, and EIPA markedly reduced PRRSV-2 replication. However, only genistein and EIPA showed no further effect in ATP1A1-knockdown cells. Moreover, ATP1A1 knockdown abolished viral-induced uptake of dextran and cholera toxin B—markers of macropinocytosis and caveolar endocytosis—confirming that these two pathways are specifically governed by ATP1A1 during PRRSV-2 entry. Activated ATP1A1-Src signaling cascade requires cholesterol, and cholesterol depletion reduces the recruitment of Src and therefore reduces ATP1A1 signaling (46). Interestingly, it has been widely reported that cholesterol-rich lipid rafts are required for PRRSV entry, and cholesterol depletion by methyl-β cyclodextrin (MβCD) has shown no effect on clathrin-mediated endocytosis of PRRSV (47–49). According to these results, the reduction in cholesterol is supposed to weaken ATP1A1-Src complex signaling in caveolae/raft, thereby reducing PRRSV entry.

PRRSV-2 invades into host cells through its envelope proteins (18). Molecular docking, co-immunoprecipitation, and GST-pulldown assays confirmed that the C-terminus of GP4 binds ATP1A1-ER4. The C-terminal domain of GP4 has been reported to function as a GPI (glycosylphosphatidyl inositol) anchor to associate GP4 to the membrane and colocalize with CD163 in the lipid rafts on the plasma membrane. Most importantly, the complete removal of the C-terminus of GP4 resulted in a complete loss of virus infectivity (50). ATP1A1 also co-localizes with PRRSV-2 in CTB-labeled caveolae/raft via binding to GP4. Hence, the interaction between ATP1A1 and the C-terminal of GP4 may be vital for determining PRRSV-2 infectivity. Notably, some viral mutations, such as GP2a-I118 and GP4-D43, which are necessary to determine viral tropism for PAMs, have been well described (51). However, the C-terminal amino acid sequence of GP4 is highly conserved among different PRRSV-2-modified live vaccines (MLVs) and wild-type strains (52), indicating that the interaction between ATP1A1 and GP4 C-terminus is generally present across PRRSV-2 strains rather than being a mechanism specifically evolved in any certain strain. As for ATP1A1-ER4, it has a dileucine-based motif “LL” and non-classical tyrosine-based motif “YEQ” known as conservative endocytic motifs (53). According to the result of molecular docking analysis, GP4 is shown to interact with the residues Q906 of ATP1A1-ER4 via hydrogen bonds, suggesting that GP4 most likely directly triggers the endocytic event of ATP1A1 via the endocytic motif on ER4. To further confirm this interaction is critical for PRRSV-2 entry, a synthetic ATP1A1-ER4 peptide was produced to detect the blocking effect on PRRSV-2 infection. As expected, ATP1A1-ER4 peptide inhibits PRRSV-2 replication via competitively reducing viral attachment and internalization in a dose-dependent manner. Moreover, a specific ATP1A1-ER4 blocker (ATP1A1-Nb) provides broad inhibition against various PRRSV-2 lineages both in PAMs and Marc-145 cells. All of these findings demonstrate ATP1A1 enhances PRRSV-2 entry and highlight the GP4–ATP1A1 interface as a promising antiviral target.

Following internalization, viral particles are transported by specific endosomes for membrane fusion (5). Here, PRRSV-2 particles were found to colocalize with Rab5-positive early endosomes, where they coexist with ATP1A1 and the canonical receptor CD163**,** suggesting that ATP1A1-related internalization delivers PRRSV-2 into early endosomal compartments that serve as platforms for subsequent uncoating and genome release. Although there is no direct interaction detected between ATP1A1 and CD163, our experiments with non-permissive cell lines further illustrate the functional interplay between ATP1A1 and CD163. In Vero cells, which express both ATP1A1 and CD163, PRRSV-2 successfully attached and internalized, whereas in PK-15 cells, which lack CD163, attachment occurred without internalization. Overexpression of CD163 in PK-15 cells restored PRRSV-2 internalization and its colocalization with ATP1A1 in cytoplasmic vesicles. These results demonstrate that ATP1A1 may act upstream of CD163: it recruits virions to the cell surface and activates signaling cascades required for uptake, subsequently mediating further viral internalization processes, whereas CD163 acts in a cooperative role. Most importantly, the combination approach blocking both ATP1A1 and CD163 could yield synergistic protection for PRRSV-2 infection in PAMs, indicating the coordinated roles of ATP1A1 and CD163 in defining viral infection. The specific cooperative mechanism between ATP1A1 and CD163 requires further research in the future.

**Fig 10:**
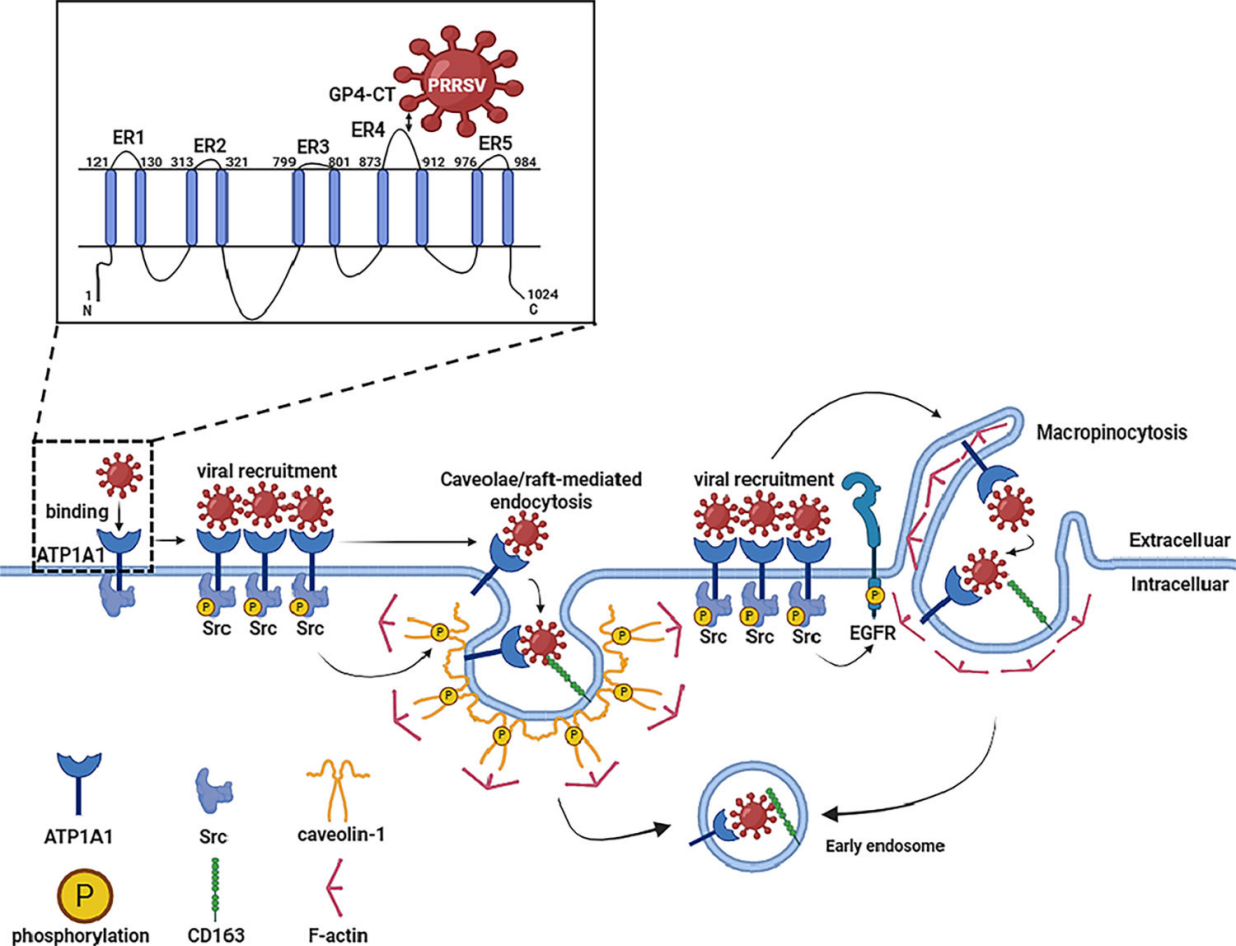
Proposed model of ATP1A1-dependent entry of PRRSV. First, PRRSV specifically recognizes and attaches to the ER4 region of ATP1A1 through GP4. Subsequently, ATP1A1 clusters to recruit more viral particles. At the same time, ATP1A1 activates downstream Src kinase activity, further promoting the phosphorylation of EGFR and Caveolin-1, thereby triggering macropinocytosis and caveolae/raft-mediated endocytosis. CD163 acts as an indispensable co-entry receptor in this process. Finally, ATP1A1, together with viral particles, enters CD163-containing early endosomes, preparing for the subsequent uncoating and genome release.

Collectively, our findings establish an uncovered model for ATP1A1-mediated PRRSV entry (Fig. 10). In this model, PRRSV engages ATP1A1 to trigger Src-dependent phosphorylation of EGFR and caveolin-1, initiating macropinocytosis and caveolae/raft-mediated endocytosis. Internalized virions are subsequently trafficked to ATP1A1/CD163-positive early endosomes, where uncoating occurs. This ATP1A1-centered signaling network integrates receptor recognition, cytoskeletal remodeling, and vesicular trafficking to orchestrate efficient viral invasion. Regretfully, ATP1A1 knockout cell lines are lethal; hence, we failed to create ATP1A1 knockout cell lines using the CRISPR-Cas9 technology. Therefore, it remains unknown whether ATP1A1 is indispensable for PRRSV-2 infectivity. However, at least, this study not only deepens understanding of PRRSV-2 pathogenesis but also provides a conceptual and experimental framework for developing next-generation antiviral agents targeting host–virus interface signaling.

## MATERIALS AND METHODS

### Cells and viruses

Marc-145, PK-15, and Vero cells (China Center for Type Culture Collection, China) were cultured in Dulbecco’s minimal essential media (DMEM; HyClone, Thermo Scientific, MA, USA) supplemented with 6% fetal bovine serum (FBS; Invitrogen, USA) at 37°C in 5% CO_2_. Primary porcine alveolar macrophages (PAMs) stored in our laboratory were grown in RPMI 1640 (Thermo) supplemented with 10% FBS at 5% CO_2_ and 37°C. The operating procedures for animal experiments to isolate PAMs were approved by the Laboratory Animal Management Committee of Zhejiang University, with number ZJU20240874.

PRRSV strains, including HuN4-F112 (GenBank ID: EF635006.1), JXA1 (GenBank ID: EF112445.1), FH (GenBank ID: EU480712.1), and JK100 (GenBank ID: AF332735.1), are stored in our laboratory. PRRSV strain HNXX16 (GenBank ID: MH588709.1) was isolated by our laboratory, and the method has been described before (54). All of the strains have been quantified by partially sequencing before the initial use. PRRSV strains were propagated and titrated in Marc-145 cells (HuN4-F112, JXA1, and JK100) or PAMs (HNXX16 and FH) and stored at −80°C.

### Viral infection

Cells were grown to approximately 70%–80% confluence and infected with the virus at the indicated MOI (dependent on different virus strains: for HuN4-F112 strain, it is based on Marc-145 cells, and for HNXX16 strain, it is based on PAMs) for 2 h at 37°C. Then, the culture medium was removed, and the cells were washed for three times, followed by incubation for indicated periods in a fresh medium containing 2% FBS at 37°C under a 5% CO_2_ atmosphere.

### Antibodies and reagents

ATP1A1 rabbit polyclonal antibody (pAb) (14418-1-AP) was purchased from Proteintech (Wuhan, China). Phospho-Src-Y419 rabbit mAb (AP1027), EGFR rabbit pAb (A11351), Phospho-EGFR-Y845 rabbit pAb (AP0023), Caveolin-1 rabbit pAb (A1555), Phospho-Caveolin-1-Y14 rabbit pAb (AP0742), rabbit anti-GST-Tag mAb (AE077), β-actin mouse mAb (AC026), and the mCherry rabbit polyclonal antibody (HA500049) was obtained from HUABIO (Hangzhou, China). The mouse anti-PRRSV N (VH13) mAb and anti-CD163 mAb (8H2) were stored in our laboratory. The FITC-labeled VH13 mAb was generated from FITC labeling kits (catalog no. C006141; Sangon Biotech, China).

Various inhibitors, chlorpromazine hydrochloride (CPZ, HY-B0407A), genistein (GEN, HY-14596), ethylisopropylamiloride (EIPA, HY-101840), PP2 (HY-13805), Src inhibitor 1 (Src-l1, HY-101053), ouabain octahydrate (ouabain, HY-B0542), and rostafuroxin (PST2238, HY-12283) were purchased from MedChemexpress.

### Virus titer detection

PRRSV titers were measured via a microtitration assay using Marc-145 cells (HuN4-F112, JXA1, and JK100) or PAMs (HNXX16 and FH) in 96-well plates. Cell culture supernatants were collected at 48 hpi to calculate 50% tissue culture-infective doses (TCID_50_) per milliliter according to the method of Reed and Muench.

### RNA interference

Cells were transfected with siRNAs targeting *ATP1A1* gene of monkey or pig at a final concentration of 75 nM via siRNA-Mate plus (GenePharma) according to the manufacturer’s instructions for 48 h. Sequences of the siRNAs are shown in Table 1. We used qRT-PCR and western blotting to detect the knockdown efficiency.

**TABLE 1:** Sequences of siRNAs

	siRNAs		Sequences
Chlorocebus sabaeus ATP1A1	si-2519	sense	GUGCAUGGUAGUGAUCUAATT
		anti-sense	UUAGAUCACUACCAUGCACTT
	si-2820	sense	GUCGUCUGAUCUUUGAUAATT
		anti-sense	UUAUCAAAGAUCAGACGACTT
	si-3285	sense	CAGCCUUCUUUGUCAGUAUTT
		anti-sense	AUACUGACAAAGAAGGCUGTT
	si-3510	sense	CCCUUCUCAUCUUCGUAUATT
		anti-sense	UAUACGAAGAUGAGAAGGGTT
porcine ATP1A1	si-320	sense	GAGGCUUCUCCAUGUUACUTT
		anti-sense	AGUAACAUGGAGAAGCCUCTT
	si-782	sense	GCACUGCACGUGGUAUUGUTT
		anti-sense	ACAAUACCACGUGCAGUGCTT
	si-1290	sense	GUCCAGAAUUGCAGGUCUUTT
		anti-sense	AAGACCUGCAAUUCUGGACTT
	si-2758	sense	AGGAAGAUCGUGGAGUUCATT
		anti-sense	UGAACUCCACGAUCUUCCUTT

### Plasmid construction and transfection

The overexpression plasmids were constructed using ClonExpress II one-step cloning kit (Vazyme, China). cDNAs encoding monkey Rab5 (GenBank ID: XM_008009261.3) and Rab7 (GenBank ID: XM_007985441.3) were obtained from Marc-145 cells cDNA. PRRSV GP4 (GeneBank ID: ABL60901.1) was amplified from PRRSV JXA1 strain. Amplified genes were subsequently subcloned and inserted into the mammalian expression vector pCMV or the fusion fluorescent expression vectors pCMV-mCherry and pCMV-eGFP. All the recombinant plasmids were verified via DNA sequencing. Primers used for plasmid construction are listed in Table 2. CD513B-CD163 overexpressing plasmid was stored in our laboratory. Cells were transfected with recombinant expression vectors via Lipofectamine 2000 (Thermo) according to the manufacturer’s instructions.

**TABLE 2:** Primers used for PCR amplification

	Primer sequences (5′–3′)	
mCherry-vector	F	GAATTCTGCAGATATGGGGATCCAGA
	R	CATGCTTCCTCCTCCTCCGC
mCherry-GP4	F	GCGGAGGAGGAGGAAGCATGGCTGCGTCCTTTCTTTTCCTCT
	R	CCCATATCTGCAGAATTCCTAGCGAATTGCCAGTAGGATGGCAA
mCherry-GP4-Mut1	F	GCGGAGGAGGAGGAAGCATGAGCTGCCTTAGGCATGGCGA
	R	CCCATATCTGCAGAATTCCTAGCGAATTGCCAGTAGGATGGCAA
mCherry-GP4-Mut2	F	GCGGAGGAGGAGGAAGCATGGCTGCGTCCTTTCTTTTCCTCT
	R	CCCCATATCTGCAGAATTCCTAATGATCGACCACTAAGGAGC
mCherry-GP4-Mut3	F	GCGGAGGAGGAGGAAGCATGGCTGCGTCCTTTCTTTTCCTCT
	R	CCATATCTGCAGAATTCCTAAGAAAGCATGAGGAGATCAGAAG
mCherry-GP4-Mut4	F	GCGGAGGAGGAGGAAGCATGGCTGCGTCCTTTCTTTTCCTCT
	R	CCATATCTGCAGAATTCCTAGATGTCCTGGAGGACAACGAAG
pGEX4T-vector	F	CGTGCCGTTCTATCTTGCTGTGCTTGTCAACGCCAGC
	R	AGAACCGCCGCCTCCAGAACCGCCACCGCCGCTACCGCCGCCACCGGATCCACGCG
pGEX4T-ER1	F	GGAACCTCAAAATGATAATTAACTTAACGTTATGTGAGCTGAATGGCAC
	R	CAGATTATCATTTTGAGGTTCCTCTTCTGTAGAACCGCCGCCTCCAGAAC
pGEX4T-ER2	F	CACCTGGCTTGAGGCTGTCTAACTTAACGTTATGTGAGCTGAATGGCAC
	R	GACAGCCTCAAGCCAGGTGTACTCAAGAGAACCGCCGCCTCCAGAAC
pGEX4T-ER3	F	CGGCGGTTCTCCACTACCATAACTTAACGTTATGTGAGCTGAATGGCAC
	R	TGGTAGTGGAGAACCGCCGCCTCCAGAAC
pGEX4T-ER4	F	GTTCTGGAGGCGGCGGTTCTCTGGCTGAGAACGGCTTCCTCC
	R	CAGCAAGATAGAACGGCACGCTCCACGATTTTCCTCTGCTC
pGEX4T-ER5	F	TTAGGATGTATCCCCTCAAATAACTTAACGTTATGTGAGCTGAATGGCAC
	R	TTTGAGGGGATACATCCTAAGAGCAACAGAACCGCCGCCTCCAGAAC
eGFP-vector	F	TCTAGAATGGTGAGCAAGGGCGAGG
	R	GAGCTCCAGCTTTTGTTCCCTT
eGFP-Rab5	F	GGAACAAAAGCTGGAGCTCATGGCTAATCGAGGCGCAAC
	R	CCTTGCTCACCATTCTAGAGTTACTACAACACTGACTCCTGGT
eGFP-Rab7	F	GGGAACAAAAGCTGGAGCTCATGACCTCTAGGAAGAAAGTGTTGC
	R	CCTTGCTCACCATTCTAGAGCAACTGCAGCTTTCTGCCG
CD163	F	TATCAAAGCCACTGGATGGGCTAAT
	R	AGGCAAGAATTCATCTCCCGGTATT

### qRT‒PCR

Total RNA was extracted to quantify the mRNA expression of target genes using qRT-PCR. Briefly, the RNA extraction kit (Easy-Do, China) was used to extract the total cell RNA, followed by a reaction of reverse transcription. qRT-PCR assay was subsequently performed as described before (55). The specific primers used for qPCR are listed in Table 3.

**TABLE 3:** Primers for quantitative PCR (qPCR) analysis

Target genes	Primer sequences (5′–3′)	
PRRSV N	F	CCTCTAGCGACTGAAGATGACGTCAGGCATCACT
	R	ACTCCACAGTGTAACTTATCCTCCCTGAATCT
Monkey β-actin	F	ATCTGGCACCACACCTTCTACAATGAGCTGCG
	R	CGTCATACTCCTGCTTGCTGATCCACATCTG
Pig GAPDH	F	CCTTCCGTGTCCCTACTGCCAAC
	R	GACGCCTGCTTCACCACCTTCT
CD163	F	TCGCTGCCAAAGAAGGACAT
	R	AGCGTTCAGACCTTCACCGT

### Western blotting

The prepared samples were electrophoresed using 12% SDS-PAGE and then transferred onto polyvinylidene difluoride (PVDF) membranes, followed by being blocked in 5% non-fat milk at 37°C for 1 h. The primary antibody was used to incubate the PVDF membranes at 4°C overnight. After three washes with TBST, the membranes were incubated with HRP-conjugated secondary antibody at 37°C for 1 h. The bands were visualized using SuperSignal West Pico/Femto chemiluminescent substrate.

### Co-IP

Co-immunoprecipitation was performed via IP lysis buffer (P0013F, Beyotime) supplemented with 1 mM phenylmethylsulfonyl fluoride (PMSF) (ST505, Beyotime). Immunoprecipitation was performed via protein A+G agarose (P2055, Beyotime) according to the manufacturer’s instructions. Briefly, protein samples were divided into two tubes. One tube, which represented the input, was denatured at 95°C for 10 min. The other mixture was first incubated with the indicated antibody at 4°C overnight, followed by incubation with 50 µL of protein A+G agarose at 4°C for 4 h. Agarose-Ab-antigen complexes were washed with ice-cold phosphate-buffered saline (PBS) five times and then resuspended in 40 µL of 1× SDS-PAGE loading buffer. Precipitates and whole-cell lysates were subjected to 12% SDS-PAGE, and the results were analyzed via western blotting.

### GST pulldown

Gene fragments ER1–ER5 of ATP1A1 were generated by PCR using monkey ATP1A1 as the template and primers shown in Table 2. Subsequently, the gene fragments were inserted into the pGEX4T plasmid containing the sequences for a GST tag. The inserted fragments were confirmed by sequencing. Plasmids were transformed into *Escherichia coli* BL21 (DE3), and protein expression was induced by 1 mM isopropyl β-D-thiogalactopyranoside (IPTG) at 16°C for 24 h and purified using GSTSep Glutathione Agarose Resin (catalog no. 20507ES10; Yeasen, China) according to the protocol. The purified recombinant GST-ER proteins were coupled to GSTSep Glutathione MagBeads (catalog no. 20562ES08; Yeasen, China) at 4°C for 2 h, where GST served as control. Then, the beads were incubated with soluble cherry-GP4 protein derived from the supernatant of HEK-293T cells lysate (transient transfection in 6-well plates) at 4°C overnight. The eluted samples were subjected to IB and detected by anti-mCherry pAb and anti-GST mAb.

### Immunofluorescence assay

For indicated protein detection, cells were first fixed with 4% PFA for 30 min at 37°C and then permeabilized with 0.2% Triton X-100 in PBS for 10 min. Notably, for the assays including ATP1A1 protein detection, pre-colded methanol was used to fix the cells. Fixed cells were washed with PBS and blocked with 10% FBS for 1 h at 37°C. Then, the cells were incubated with indicated primary antibodies at 4°C overnight, followed by secondary antibody incubation at 37°C for 1 h. The nuclei were stained with DAPI (1:2,000 diluted in PBS). For some confocal microscopy, GFP-fused ATP1A1-Nb (1:200 diluted in PBS) and FITC-labeled VH13 mAb (1:200 diluted in PBS) were used to stain ATP1A1 and PRRSV N after the incubation of secondary Alexa Fluor-conjugated IgG (H+L). The cells were observed by fluorescence microscope.

### Virus attachment and internalization assay

For viral attachment assay, PRRSV HuN4-F112 strain (MOI = 50) infected cells at 4°C for 2 h. Then the cells were washed with cold PBS for six times, and cell samples were collected and detected. For viral internalization assay, PRRSV HuN4-F112 strain (MOI = 50) infected cells at 4°C for 2 h. Then, the cells were washed with cold PBS for six times and transferred to 37°C for 2 h incubation to complete virus internalization. Then, the cells were washed six times with acidic PBS (pH = 2.0) to remove the uninternalized virus particles. After that, cell samples were collected and detected.

### Time-of-drug-addition experiment

Marc-145 cells grown in 48-well plates were separately treated with ouabain (25 nM) and PST2238 (25 µM) at the indicated times: pre-2 h, 0 h, post-2 h, post-4 h, and post-8 h (all of groups with compounds present throughout). DMSO is used as a negative control. For the 2 h treatment, cells were pretreated with drugs for 2 h at 37°C prior to virus infection. After pre-treatment, cells were infected with the PRRSV HuN4-F112 strain (MOI = 1) at 37°C for 2 h. Then, the supernatant was removed, and the cells were washed with Hanks’ balanced salt solution and incubated in the presence of fresh medium containing 2% FBS at 37°C. For the 0 h treatment, PRRSV and drugs were added to the cells simultaneously and incubated at 37°C for 2 h. Following infection, the supernatant was removed, cells were washed with Hanks’ balanced salt solution, and culture medium was replaced with DMEM containing 2% FBS. For post-infection treatments, cells were first infected with PRRSV at 37°C for 2 h. After removal of the supernatant and washing with Hanks’ balanced salt solution, DMEM containing 2% FBS was added. Drugs were subsequently added at 2, 4, or 8 hpi, respectively. In each case, the culture medium contained the drugs throughout. All of the cell samples were collected at 10 hpi to detect viral RNA abundance via qRT-PCR.

### Endocytic marker uptake assay and flow cytometry

Endocytic markers, including FITC-labeled Transferrin (TFN-FITC, 10 µg/mL, Cat. No. AB_2337074; Jacson ImmunoResearch, USA), FITC-Dextran (MW 10,000) (DTN-FITC, 200 µg/mL, Cat. No. HY-128868; MedChemexpress, USA), and Alexa Fluor 488-conjugated cholera toxin subunit B (CTB-AF488, 10 µg/mL, Cat. No. 131096-89-4; Absin, China), were used to incubate Marc-145 cells for 1 h. Then, the cells were uninfected or infected with the PRRSV HuN4-F112 strain (MOI = 50). At 1 hpi, cells were then washed with DMEM three times to remove residual staining solution. Green fluorescence was detected using BD FACSVerse (BD Biosciences, USA) through FITC channel. FlowJo software (v10.8.1) was used to analyze the data.

### Peptide blocking assay

The amino acid sequences of ATP1A1-ER peptides are listed in Table 4. To verify the function of GST-ER peptides on PRRSV infection, 200 TCID50 of PRRSV HuN4-F112 was mixed with peptides at various concentrations at 37°C for 2 h. After incubation, 100 µL of the peptides-virus mixture was added to Marc-145 cells in each well of 96-well (for IFA detection) or 48-well (for qRT-PCR detection) plate at 37°C for 2 h. Then, the supernatants were removed, and the cells were rinsed three times with Hanks’ balanced salt solution and incubated in the presence of fresh medium containing 2% FBS at 37°C under a 5% CO_2_ atmosphere. At 24 hpi, cell samples were collected, and virus replication was identified in IFA and qRT-PCR. In this assay, purified GST protein was used as the negative control.

**TABLE 4:** Amino acid sequences of ATP1A1 extracellular domains

Target genes	Sequence
ATP1A1-ER1	TEEEPQNDNL
ATP1A1-ER2	LEYTWLEAV
ATP1A1-ER3	PLP
ATP1A1-ER4	LAENGFLPLHLLGLRVDWDDRWINDVEDSYGQQWTYEQRK
ATP1A1-ER5	VALRMYPLK

### Pre-entry and post-entry assay

For pre-entry assay, the GST-ER4 peptide (100 μg/mL) was incubated with PRRSV HuN4-F112 (200 TCID_50_) at 37°C for 2 h. Then, the mixture was added to single-layer Marc-145 cells and replaced with DMEM medium containing 2% FBS after 2 h incubation. At 24 hpi, virus replication was detected by qRT-PCR, and viral titers were determined by detecting TCID_50_ at 48 hpi. For post-entry assay, Marc-145 cells were infected with PRRSV HuN4-F112 (200 TCID_50_) at 37°C for 2 h. Then, the cells were inoculated by GST-ER4 peptide (100 μg/mL), followed by washing three times with PBS after 2 h incubation. After this, the peptide-containing medium was replaced with DMEM containing 2% FBS. At 24 hpi, virus replication was detected by qRT-PCR, and viral titers were determined by detecting TCID_50_ at 48 hpi. In this assay, purified GST protein was used as the negative control.

### GST-ER4-targeted polyclonal antibody and nanobody inhibition assay

Mice were immunized with GST-ER4 peptide or GST only protein to produce polyclonal antibodies (pAbs). Marc-145 cells on 48-well plates were incubated with GST-ER4 pAb in a 100-fold dilution in DMEM at 37°C for 2 h. GST pAb was used in parallel as a control. Cells were then infected with PRRSV HuN4-F112 (200 TCID_50_) at 37°C for 2 h. After three washes, cells were continually incubated with DMEM containing the corresponding antibodies at 37°C. At 24 hpi, virus replication was detected by qRT-PCR, and viral titers were determined by detecting TCID_50_ at 48 hpi.

A nanobody (ATP1A1-Nb) specifically targeting ATP1A1-ER4 was screened according to the phage display technology described in our previous study (38). Marc-145 cells or PAMs on 48- or 96-well plates were incubated with ATP1A1-Nb (50 μg/mL) at 37°C for 2 h. PBS treatment was designed as the negative control. The cells were then infected with PRRSV JXA1 and JK100 strains in Marc-145 cells and with PRRSV HNXX16 and FH strains (200 TCID_50_) in PAMs at 37°C for 2 h. After three washes, cells were continually incubated with DMEM/RPMI 1640 containing ATP1A1-Nb at 37°C. At 24 hpi, virus replication was detected by qRT-PCR and IFA, and viral titers were determined by detecting TCID_50_ at 48 hpi.

### Cell viability and cytotoxicity assay

Cell viability was determined by CCK-8 assay. Briefly, Marc-145 cells or PAMs were seeded into 96-well plates. The old culture medium was then replaced with fresh culture medium containing different concentrations of indicated chemical inhibitors or ATP1A1-Nb. At indicated hour-post-addition, 10 µL of CCK-8 reagent (CCK-8; Beyotime Institute of Biotechnology, Shanghai, China) was added to each well and incubated at 37°C for 2 h. The absorbance of each well at 450 nm was detected using a microplate reader.

### F-actin polymerization assay

The viral internalization assay was performed in drug-treated or siRNA-transfected Marc-145 cells; cells were fixed and washed three times with PBS and incubated with Actin-Tracker Green-488 (catalog no. C2201S; Beyotime, China) for 1 h (200-fold dilution). Then, the F-actin polymerization in cells was analyzed by performing a confocal laser scanning microscopy assay.

### Prediction of protein‒protein complex structure

The 3D model of the protein was constructed via Alphafold 3 (https://alphafoldserver.com/) and then visualized via PyMOL 2.5 software.

### Statistical analysis

Data are presented as means ± standard errors. All the statistical analyses were performed using GraphPad Software. Significant differences compared to controls were determined at thresholds of *P < 0.05, **P < 0.01, ***P < 0.001, and ****P < 0.0001.

## Data Availability

Relevant data have been stored at https://doi.org/10.6084/m9.figshare.31437277.
